# Sound‐offset encoding is related to speech‐in‐noise perception at sentence level in older adults

**DOI:** 10.1113/JP289104

**Published:** 2025-12-16

**Authors:** Hasan Colak, Xiaoxuan Guo, Adèle Simon, Ester Benzaquén, William Sedley, Jennifer F. Linden, Timothy D. Griffiths

**Affiliations:** ^1^ Biosciences Institute Newcastle University Newcastle upon Tyne UK; ^2^ Department of Audiology Hacettepe University Ankara Türkiye; ^3^ Ear Institute University College London London UK; ^4^ Translational and Clinical Research Institute Faculty of Medical Sciences Newcastle University Newcastle Upon Tyne UK; ^5^ Department of Neuroscience, Physiology, & Pharmacology University College London London UK; ^6^ Department of Imaging Neuroscience University College London London UK; ^7^ Human Brain Research Laboratory University of Iowa Iowa City Iowa USA

**Keywords:** auditory processing, sound offset, sound onset, speech‐in‐noise, temporal processing

## Abstract

**Abstract:**

Sound‐onset and sound‐offset encoding are critical aspects of auditory temporal processing with important roles in speech‐in‐noise perception. We investigated brain correlates of active‐offset encoding in older adults and their relationship to real‐world listening. A total of 34 adults aged 60–81 years underwent pure‐tone audiometry and assessments of speech‐in‐noise ability at both word and sentence levels. EEG data were recorded while participants performed active duration discrimination of noise segments and during passive listening. Onset and offset responses were extracted from EEG data, and amplitude differences were analysed. We found robust onset and offset responses at a single‐subject level. Onset responses were significantly reduced in the active condition compared to passive listening, whereas offset responses were significantly larger. This pattern suggests that offset encoding is more susceptible to attentional modulation, whereas onset responses may be less affected. We found that active sound‐offset amplitudes were significantly correlated with sentence‐in‐noise performance, whereas no correlation was observed for word‐in‐noise performance. We suggest a role for offset analysis in parsing of the speech stream needed for speech segregation. The active EEG measure we use allows single‐subject inference and is a potential clinical measure of auditory cognition relevant to speech‐in‐noise listening.

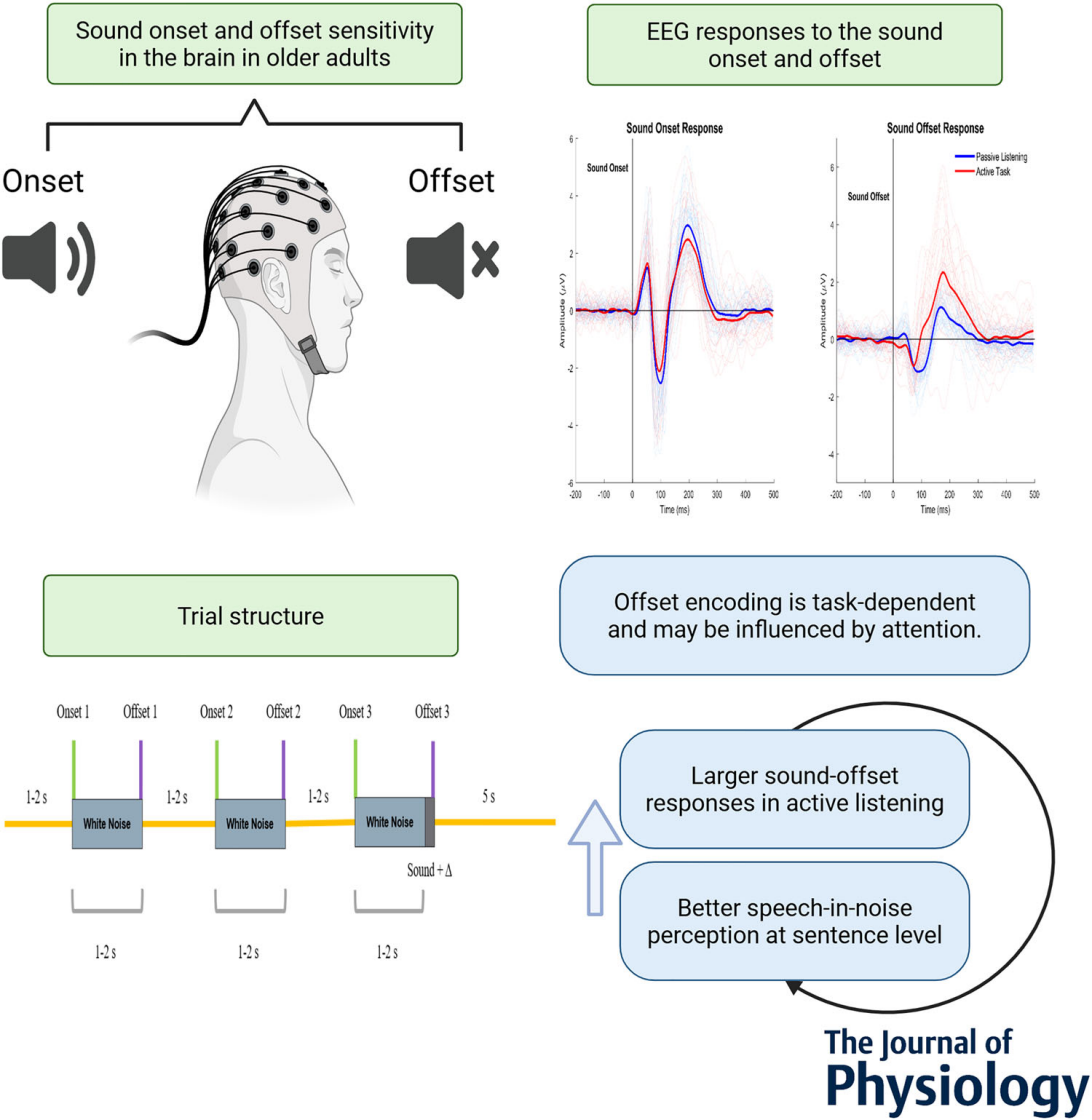

**Key points:**

- Sound‐offset responses can be robustly identified at the single‐subject level using EEG.
- Sound‐offset responses were larger in the active task than in passive listening, suggesting task dependency and indicating that attention may be involved in sound‐offset encoding.
- Active sound‐offset responses correlate with sentence‐level speech‐in‐noise performance.

## Graphical abstract

**Abstract figure legend** Schematic summary of the study investigating sound‐onset and offset sensitivity in the brain of older adults. EEG responses to white‐noise bursts were recorded to examine neural encoding of sound onset and offset during passive listening and active task conditions. Offset responses were found to be task‐dependent and influenced by attention, showing larger amplitudes during active listening. Importantly larger active offset amplitudes were associated with better sentence‐in‐noise performance. These findings suggest that offset encoding reflects attention‐sensitive neural processes that support speech stream parsing and real‐world speech‐in‐noise understanding in ageing listeners.

## Introduction

Humans live in a dynamic auditory environment in which sounds constantly change, often without any predictable pattern. This chaotic nature of our surroundings emphasizes the importance of efficiently processing acoustic cues that are critical for survival, communication and navigating social interactions. Among these cues the ability to encode sound onsets and offsets plays a crucial role in perceiving and interpreting external auditory stimuli.

Historically offset responses have been less studied compared to onset responses. Onset units are more prevalent and have a well‐organized tonotopic representation compared to offset units (Baltzell & Billings, 2014). Moreover abrupt increases in sound intensity, such as those associated with onset responses, are more commonly encountered in the natural world than abrupt decreases in sound intensity, which define offset responses (Kopp‐Scheinpflug et al., 2018). Although the exact mechanism of sound‐offset encoding remains unknown, the most popular view is that sound‐offset responses are driven by the interaction between excitatory and inhibitory inputs (Kopp‐Scheinpflug et al., 2018, 2011). More specifically offset responses may be generated by a dedicated sound‐offset pathway in the auditory brain, ranging from the cochlear nucleus to the auditory cortex, in which inhibitory transmission likely plays a primary role in offset response generation (Kasai et al., 2012; Kopp‐Scheinpflug et al., 2018; Qin et al., 2007). At the cortex level both onset and offset responses are driven by transient synaptic inputs, but they typically arise from largely nonoverlapping sets of synapses with different frequency tuning, indicating that onset and offset responses are partly distinct yet coexisting mechanisms within the auditory cortex (Scholl et al., 2010).

The earlier attempts to record EEG onset and offset responses in the human brain have revealed asymmetries between these responses, such as smaller and slower offset responses compared to onset responses (Hillyard & Picton, 1978; Pantev et al., 1996). Hillyard and Picton (1978) also reported that sound‐offset responses depend on stimulus duration, with offset amplitudes increasing as the duration becomes longer. Offset EEG responses show morphological differences compared to onset responses. For example the P1 component is consistently absent in offset responses, whereas onset responses typically follow the P1–N1–P2 waveform (Pantev et al., 1996; Pratt et al., 2008). At the cortical level offset responses are generated predominantly in the bilateral auditory cortex on the supratemporal plane, particularly in Heschl's gyrus and the planum temporale, similar to onset responses (Hari et al., 1987; Pantev et al., 1996). Pratt et al. (2005) studied the effect of attention on the sound‐offset response and found no evidence of attentional modulation in the earlier N1 component. Rather Harris et al. (2012) reported an attentional modulation in the later N2 component of the sound‐offset response. However many previous studies overlooked the role of stimulus duration, which may have led to interference between onset and offset responses, making it difficult to clearly separate the two.

Previous work on the ecological and clinical relevance of sound offsets has suggested that they are important for many perceptual aspects of hearing, including processing temporal fluctuations, detecting silent gaps in continuous sounds, encoding the duration of sounds, and temporal integration (Ali et al., 2025; Anderson & Linden, 2016; Li et al., 2021; Solyga & Barkat, 2021; Song et al., 2024). Categorical perception of speech sounds also involves offset mechanisms: offset detection is needed to define voice onset times that are critical to consonant discrimination (Kopp‐Scheinpflug et al., 2018). Ageing is well known to degrade temporal resolution in the auditory system (Anderson & Karawani, 2020; Strouse et al., 1998), even when controlling for age‐related hearing loss (Füllgrabe et al., 2014; Heidari et al., 2023). Poor temporal encoding might affect speech understanding in noise because speech‐in‐noise listening often requires precise temporal encoding of acoustic cues to parse overlapping or transient speech signals (Anderson et al., 2022, 2010; Gordon‐Salant & Fitzgibbons, 1993; Petley et al., 2024). However the specific aspects of temporal encoding that contribute to difficulties in understanding speech in noise remain poorly understood. Both the onset and offset components of temporal encoding are likely to be involved, as they enable listeners to detect, segment and interpret acoustic cues amidst competing background noise. Age‐related degradation of these onset and offset mechanisms may help explain why older adults often struggle to understand speech in noisy environments.

The aims of this study are to determine whether brain responses to sound offsets can be reliably recorded at the single‐subject level in older adults and to compare them with onset responses. Our goal is to understand whether offset responses are robust in this population and to pave the way for potential clinical applications and further investigation in auditory cognitive disorders. We also aim to assess whether onset and offset responses are modulated by task demands by comparing passive listening with an active duration discrimination task, allowing us to test the role of attention. Finally we investigate the relevance of onset and offset responses to everyday listening by examining their relationship with word‐ and sentence‐in‐noise performance in older adults.

## Methods

### Ethical approval

The experimental procedure was approved by the Newcastle University Research Ethics Committee, and written informed consent was obtained from all participants (reference number 46,225/2023). The study conformed to the standards set by the Declaration of Helsinki (as revised in October 2013), except for registration in a database.

### Participants

Forty older adults (aged ≥ 60 years) with no worse than mild hearing loss were recruited for the study. Six subjects were excluded, with four removed due to excessive EEG artefacts and two because of incomplete data. This resulted in a final sample of 34 participants (23 female) aged between 60 and 81 years (*n* = 34). Exclusion criteria included the use of psychotropic drugs and a history of mental health or neurological conditions (e.g. autism, dyslexia). All participants were native English speakers.

All participants underwent pure‐tone audiometric evaluation in the 0.25–8 kHz range, following the modified Hughson‐Westlake procedure. Pure‐tone averages (PTA) were calculated by averaging the hearing thresholds across the 0.25–8 kHz audiometric range. To ensure that the auditory stimuli were audible participants were included only if they had normal hearing or no more than mild hearing loss (PTA < 40 dB HL). Audiograms for all participants are shown in Fig. 1. All tests were conducted while participants were seated in a comfortable chair in a soundproof and electrically shielded room. Custom‐written computerized auditory tests were performed using Sennheiser HD 201 circumaural headphones, and the stimuli were presented using Psychtoolbox in MATLAB 2017a. Practice trials were provided for all computer tasks to familiarize participants with the tasks and stimuli.

**Figure 1:**
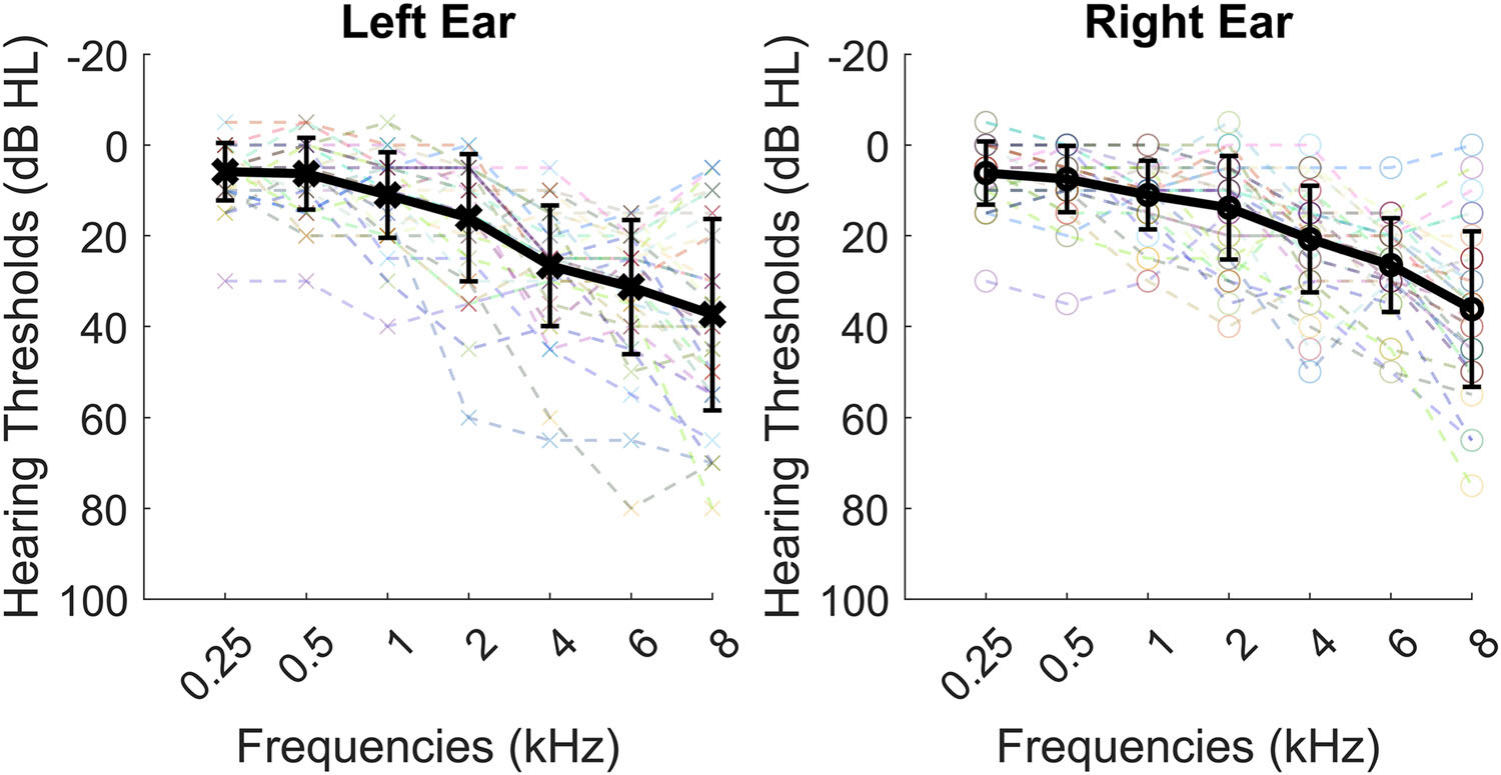
Pure‐tone audiometry thresholds of the participants for the left and right ears Individual audiogram data are represented by coloured lines, whereas black lines indicate the averaged data. Error bars represent one standard deviation.

### Materials

#### Word‐in‐noise test

The British version of the Iowa Consonant Perception Test was used to assess single‐word‐level speech perception in noise (Guo et al., 2024). The test featured one female and one male speaker, both with British Southern Standard accent. The material consisted of 120 trials, where participants were presented with four phonetically balanced alternative words on the screen and asked to select the target word from the options. The target stimuli were monosyllabic words with a consonant‐vowel‐consonant structure (e.g. ‘ball‐fall‐shawl‐wall’). An eight‐talker babble noise was used as the background noise, and stimuli were presented at a constant signal‐to‐noise ratio (SNR) of −2 dB throughout the test. The target words were presented at a sound level of 70 dB SPL. The outcome measure was the percentage of correctly identified target words among the 120 trials.

#### Sentence‐in‐noise test

The sentence‐in‐noise test was implemented using JavaScript. We used the English version of the Oldenburg sentences as the target speech material, which followed the <Name> <Verb> <Number> <Adjective> <Noun> structure (Holmes & Griffiths, 2019). Sentences were presented with 16‐talker babble noise, and participants were shown a 5 × 10 matrix, where 10‐word options were provided for each word category. Participants were asked to select the words they heard from the given options. The test followed a one‐up one‐down procedure, starting at a 10 dB SNR level and presented at 70 dB SPL. The target speech was kept at a constant intensity level, whereas the noise intensity varied based on participants' performance. The step sizes decreased from 3 to 2 dB after three reversals, which then reduced to 1 dB after three more reversals. Step size for the last six reversals was 0.5 dB SNR. The run terminated after 12 reversals, and the median of the last six reversals was used to calculate final dB SNR threshold for each participant. Lower scores indicated better performance on the test.

#### Gap‐detection test

We used a gap‐detection test (GDT), as detecting the silent gap in a continuous sound may involve the brain's sound‐offset encoding mechanism (Anderson & Linden, 2016). The test employed a three‐alternative forced‐choice design. A 500 ms white noise without an onset ramp was presented, and the silent gap was randomly placed between 100 and 400 ms. The gap duration varied between 2 and 30 ms. The test followed a two‐down one‐up procedure with a total of 15 reversals. The average of the last five reversals was used to calculate the gap‐detection threshold.

### EEG experimental design

EEG responses were recorded both under passive listening condition and during a duration discrimination task. Because there is no validated measure of sound‐onset and offset sensitivity we implemented a duration discrimination design that engages both sound‐onset and offset encoding to detect sound duration differences. In the duration discrimination task participants were presented with three white‐noise stimuli without ramps and were asked to say whether the longest sound was the first, second or third. This design enabled the recording of three sound‐onset and three sound‐offset response components per trial. The duration of each sound varied randomly between 1 and 2 s across trials to allow recovery from sound‐onset responses and to prevent prediction‐driven differences in responses. Similarly the interstimulus interval (ISI) was randomized between 1 and 2 s to avoid predictability effects. Trials were separated by a 5 s silent period to provide sufficient time for neural recovery and to minimize the effects of repetition suppression.

For the active duration discrimination task we employed a three‐down, one‐up adaptive tracking procedure. The total number of reversals was set to 12, and the average of the last five reversals was calculated to determine the behavioural threshold. We did not use a fixed number of trials across participants, as trials near or far from the threshold might lead to amplitude differences in onset and offset responses. Therefore the number of trials varied between participants, ranging from a minimum of 40 to a maximum of 70. A schematic for the active duration discrimination task is shown in Fig. 2. The same design was used for the passive listening condition, during which participants watched a silent movie instead of responding to the sounds. They were instructed to ignore the sounds and focus on the movie. For the passive listening condition the number of trials was fixed at 60 for all participants, resulting in 180 sound‐onset and 180 sound‐offset responses per individual. Participants still heard one sound that was longer than the other two sounds, but as this was a passive listening task the extra duration added to the longest sound was randomized between 50 and 500 ms. The passive condition was always followed by the active one across participants. Stimulus intensity was kept at 75 dB SPL throughout the experiment.

**Figure 2:**
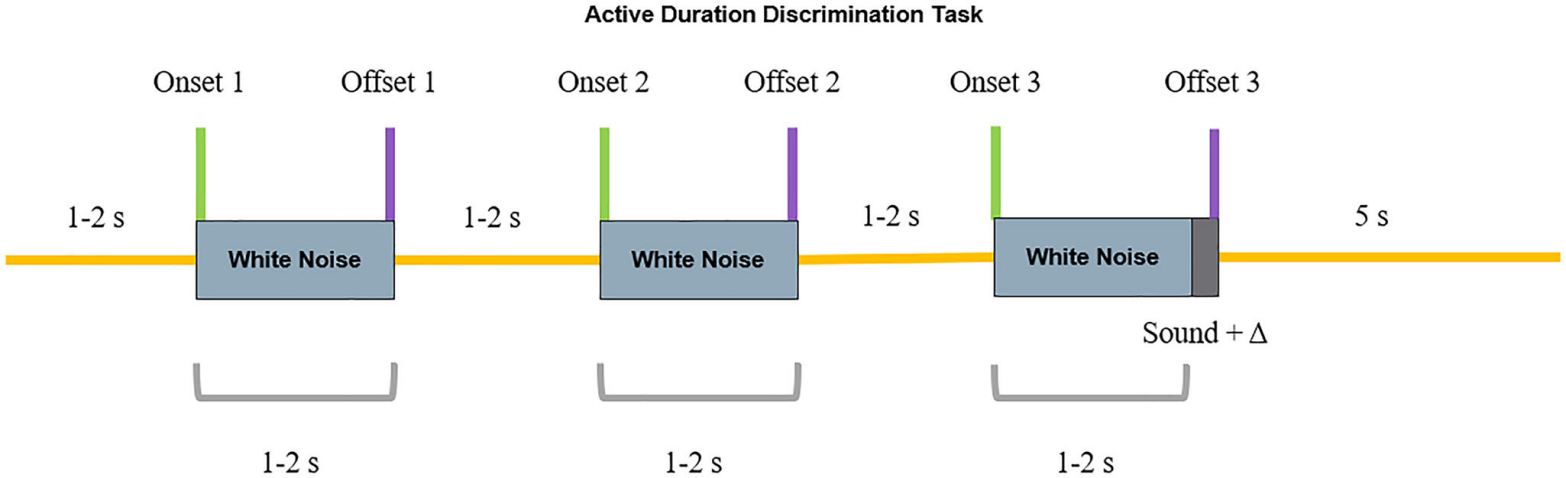
Example trial structure of the active listening task This figure illustrates the trial structure of the active duration discrimination task, used to investigate sound‐onset and offset sensitivity. Participants were presented with three consecutive white‐noise stimuli (indicated in blue), allowing us to record three onset and three offset responses (indicated in green and purple, respectively) per trial. Participants were asked to identify the sound that differed in duration. The presented white‐noise stimuli each had a duration randomly varying between 1 and 2 s (shown in grey). Orange lines indicate the silent periods. Each trial ended with a 5 s silent period to allow sufficient time for neural recovery. In the passive listening condition the same design was applied, but participants watched a silent movie instead of responding to the sounds.

### EEG data processing

The data were recorded using a 64‐channel Biosemi EEG set‐up. Data analysis was performed in MATLAB using the EEGLAB toolbox (Delorme & Makeig, 2004). We re‐referenced the data to the combined P9/P10 electrodes, which roughly correspond to the mastoid area. EEG data were first filtered with a high‐pass filter at 0.1 Hz, followed by a low‐pass filter at 30 Hz. The data were recorded at a 1024 Hz sampling rate and then downsampled to 256 Hz during preprocessing. Bad channels were removed using artefact subspace reconstruction (ASR) with a 0.8 correlation cut‐off relative random sample consensus (RANSAC). The removed channels were interpolated, and participants with more than eight removed channels were excluded from further analysis. Four participants had more than eight removed channels and were thus excluded from the study. After interpolation the data were re‐referenced to the common average. The data were epoched from −0.2 to 0.5 s relative to sound‐onset and offset events. N1–P2 responses were identified as negative and positive deflections occurring within the 50–180 ms (N1) and 120–300 ms (P2) time windows, respectively, following the onset and offset of the sound stimulus. Based on the data from Ali et al. (2025) and pilot subjects in this study FCz was selected for further analysis because it showed the largest onset and offset amplitudes. Baseline correction was applied to the epochs between −0.2 and 0 s. The onset/offset (on/off) ratio was calculated to investigate whether differences in onset and offset encoding within the same participant could be more sensitive in detecting onset/offset mechanism‐related changes.

### Statistical analysis

We used MATLAB to perform the statistical analyses. To determine whether sound‐offset responses were significantly identifiable for each participant in both passive and active listening we also conducted one‐tailed *t* tests to assess whether N1–P2 amplitudes significantly deviated from zero. False discovery rate (FDR) using the Benjamini–Hochberg method was then applied to account for multiple comparisons. Paired‐sample permutation *t* tests were conducted to compare onset and offset amplitudes between passive and active listening conditions. Onset and offset peak‐to‐peak amplitude values were shuffled 1000 times under a two‐tailed null hypothesis to generate the permutation distribution (Groppe et al., 2011). The reported *P*‐values were corrected for multiple comparisons across the contrasts using the Benjamini–Hochberg procedure with the FDR set at 0.05. Cohen's dz was calculated as a measure of within‐subject effect size to quantify the differences between conditions. We assessed whether the data distribution violated normality using the Shapiro‐Wilk test. Because the onset and offset EEG data were non‐normally distributed we used Spearman's correlation coefficient to evaluate the relationship between onset and offset amplitudes and speech‐in‐noise tests. We report both uncorrected (p) and FDR‐adjusted (*q*) values (the latter across 30 correlations), using the Benjamini–Hochberg method. FDR correction was implemented using the fdr_bh function in MATLAB. To explore whether these correlations were independent of other variables such as PTA partial correlation analyses were performed.

## Results

Thirty four participants without any neurological or psychiatric history were included in the analysis. The age of the participants ranged from 60 to 81 years, with a mean value of 68.47 years and a standard deviation of 5.05 years. Further descriptive statistics are shown in Table 1.

**Table 1:** Descriptive statistics of the participants

	Mean	Standard deviation
GDT	4.28 (ms)	1.24 (ms)
DDT	256.92 (ms)	148.88 (ms)
Passive onset	6.16 (µV)	1.84 (µV)
Passive offset	2.65 (µV)	0.66 (µV)
Active onset	5.25 (µV)	1.87 (µV)
Active offset	3.88 (µV)	1.43 (µV)
On/Off active	1.48 (µV)	0.74 (µV)
WIN	59 (%)	6 (%)
SIN	−0.91 (dB SNR)	1.40 (dB SNR)
PTA	18.56 (dB HL)	7.66 (dB HL)

*Note*: Mean and standard deviation values are shown for each behavioural and electrophysiological variable. Passive and active onset and offset amplitudes represent EEG‐derived N1–P2 peak‐to‐peak measures.

Abbreviations: DDT, duration discrimination task; GDT, gap detection test; PTA, pure‐tone average hearing threshold across 0.25–8 kHz; SIN, sentence‐in‐noise test; WIN, word‐in‐noise test.

We calculated N1–P2 amplitudes and then ran one‐tailed *t* tests for sound‐offset responses to determine whether offset responses were identifiable at the single‐subject level. All participants showed significant sound‐offset responses for both passive and active listening even after correction for multiple comparisons (*P* < 0.001; see Supplementary Material 1). We compared N1–P2 amplitudes across the passive and active listening conditions for both sound‐onset and offset responses using a permutation analysis. Individual and grand average onset and offset responses are shown in Fig. 3. In both passive and active listening conditions N1–P2 sound‐onset responses had significantly larger amplitudes than maximal sound‐offset responses (passive: *t*(33) = 11.355, *P* < 0.001, Cohen's dz = 1.95; active: *t*(33) = 4.108, *P* = 0.001, Cohen's dz = 0.71). We found significantly larger sound‐onset responses in the passive listening condition than in the active listening condition (*t*(33) = 4.011, *P* = 0.001, Cohen's dz = 0.69). Sound‐offset responses were significantly larger in the active listening condition compared to the passive listening condition (*t*(33) = 5.431, *P* < 0.001, Cohen's dz = 0.93). Figure 4 shows the mean amplitudes of N1–P2 responses to sound onset and offset for both listening conditions.

**Figure 3:**
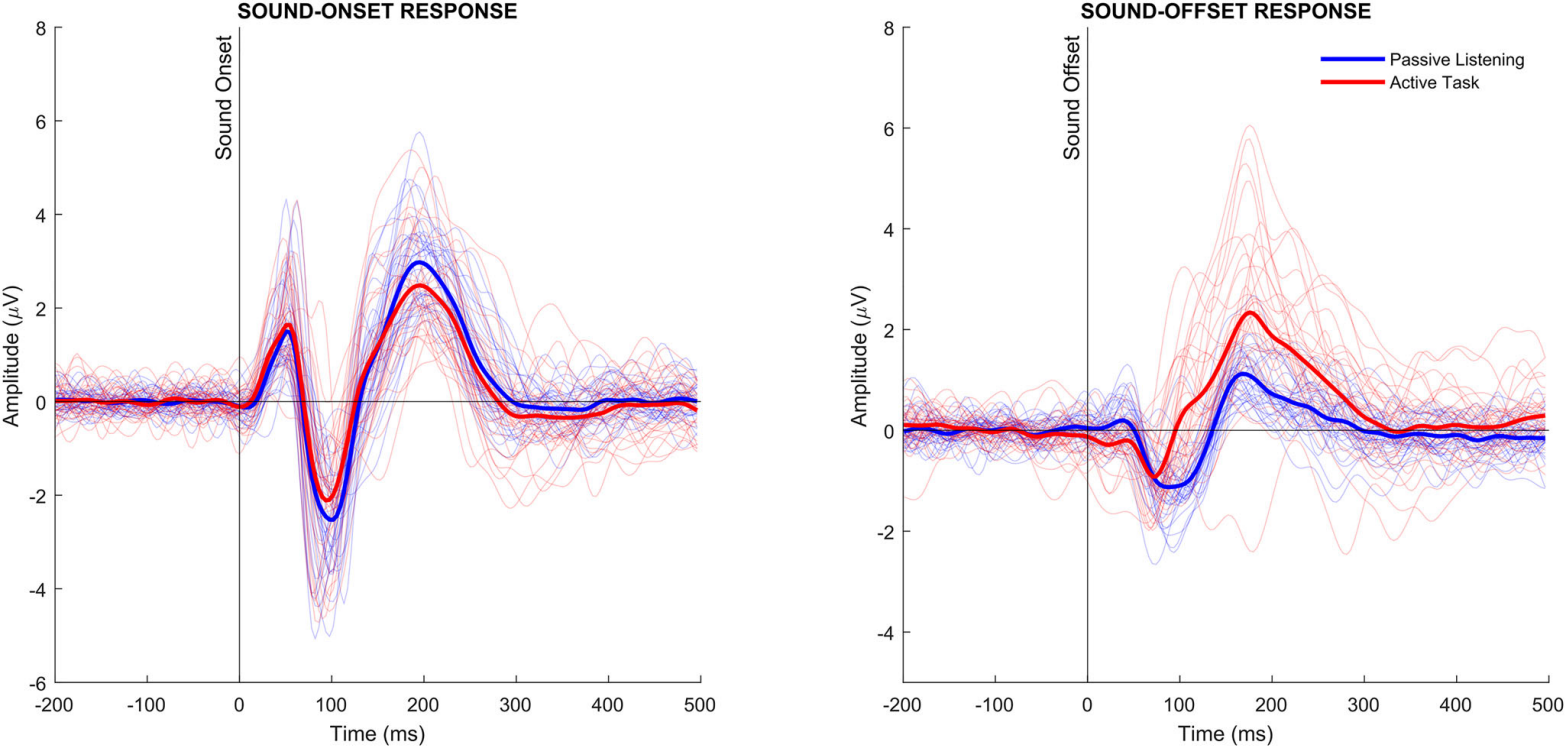
Sound‐onset (left) and offset (right) responses recorded from participants Thinner lines represent individual data, whereas thicker lines indicate the grand averaged responses. Blue lines represent the passive listening condition, and red lines indicate the active listening condition.

**Figure 4:**
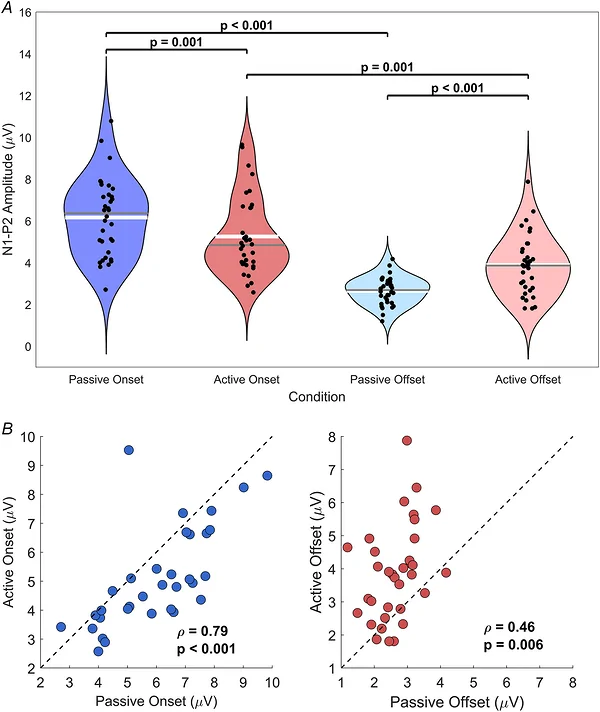
*A* N1–P2 amplitudes (µV) for sound‐onset and offset responses under passive and active listening conditions Violin plots illustrate the distribution of amplitude values for each condition. Blue and light blue violins represent the passive listening condition for onset and offset responses, respectively, whereas red and pink violins depict the active listening condition for onset and offset responses. The black dots represent individual data points for each condition. The white line within each violin indicates the mean amplitude, whereas the grey line shows the median amplitude. The width of the violin reflects the density of data points at different amplitude levels, with wider sections indicating a higher concentration. ***B* The scatterplots show the association between active and passive listening for onset (left) and offset (right) responses**. A dashed line (slope = 1) is included in each plot as a reference for active and passive responses. The Spearman correlation coefficients are shown within each plot.

Given our interest in sound‐onset and offset encoding and its relationship with behavioural measures, including speech‐in‐noise performance, we conducted correlation analyses. Table 2 shows the Spearman's rho (ρ) between behavioural and EEG onset/offset responses with the significance level after applying the Benjamini–Hochberg FDR correction for multiple comparisons. We had prior hypotheses about correlation between the active EEG condition with gap detection, duration discrimination, and the two speech‐in‐noise measures. Examination of the specified correlations revealed significant negative correlation between the active EEG offset response and sentence‐in‐noise performance threshold with a large effect size (ρ = −0.58, *P* < 0.001, *q *= 0.010). Note that the negative correlation means a relationship between larger active EEG‐offset responses and better sentence‐in‐noise performance (lower sentence‐in‐noise threshold). There was also significant correlation without correction between active EEG onset and sentence‐in‐noise performance (ρ = −0.41, *P* = 0.016, *q* = 0.096). Examination of all correlations at the uncorrected level also demonstrated correlation between passive‐offset responses and both word‐in‐noise and sentence‐in‐noise measures (ρ = 0.41, *P* = 0.016, *q* = 0.096; ρ = −0.42, *P* = 0.014, *q* = 0.096) that were not significant at the corrected level. Figure 5 illustrates the scatter plot correlations for the speech‐in‐noise tests and sound‐onset and offset responses in the active listening condition.

**Table 2:** Spearman's correlations between electrophysiological measures and behavioural performance

	Active offset	Active onset	Passive offset	Passive onset	On/off active	Active/passive offset ratio
GDT	ρ = 0.03 *P* = 0.862 *q* = 0.862	ρ = −0.09 *P* = 0.632 *q* = 0.732	ρ = −0.19 *P* = 0.288 *q* = 0.598	ρ = −0.25 *P* = 0.160 *q* = 0.494	ρ = −0.03 *P* = 0.854 *q* = 0.862	ρ = 0.17 *P* = 0.330 *q* = 0.611
DDT	ρ = −0.13 *P* = 0.460 *q* = 0.732	ρ = −0.25 *P* = 0.148 *q* = 0.494	ρ = −0.19 *P* = 0.269 *q* = 0.598	ρ = −0.24 *P* = 0.171 *q* = 0.494	ρ = −0.09 *P* = 0.615 *q* = 0.732	ρ = 0.08 *P* = 0.659 *q* = 0.732
WIN	ρ = 0.17 *P* = 0.347 *q* = 0.611	ρ = −0.08 *P* = 0.659 *q* = 0.732	ρ = 0.41 *P* = 0.016 *q* = 0.096	ρ = −0.18 *P* = 0.299 *q* = 0.598	ρ = −0.22 *P* = 0.221 *q* = 0.553	ρ = −0.09 *P* = 0.623 *q* = 0.732
SIN	**ρ** **= **−**0.58** ***P* < 0.001** *q* **= 0.010**	ρ = −0.41 *P* = 0.016 *q* = 0.096	ρ = −0.42 *P* = 0.015 *q* = 0.096	ρ = −0.25 *P* = 0.156 *q* = 0.494	ρ = 0.06 *P* = 0.720 *q* = 0.771	ρ = −0.23 *P* = 0.181 *q* = 0.494
PTA	ρ = −0.09 *P* = 0.597 *q* = 0.732	ρ = ‐0.32 *P* = 0.064 *q* = 0.321	ρ = −0.45 *P* = 0.008 *q* = 0.096	ρ = −0.09 *P* = 0.627 *q* = 0.732	ρ = −0.11 *P* = 0.528 *q* = 0.732	ρ = 0.12 *P* = 0.514 *q* = 0.732

*Note*: Spearman's rank correlation coefficients (ρ) are shown with uncorrected *P*‐values and false discovery ratio (FDR)‐adjusted *q*‐values for correlations between behavioural measures (rows) and EEG onset/offset responses (columns). Significant correlations that survived correction are indicated in bold.

Abbreviations: DDT, duration discrimination task; GDT, gap detection test; PTA, pure‐tone average (0.25–8 kHz); SIN, sentence‐in‐noise test; WIN, word‐in‐noise test.

**Figure 5:**
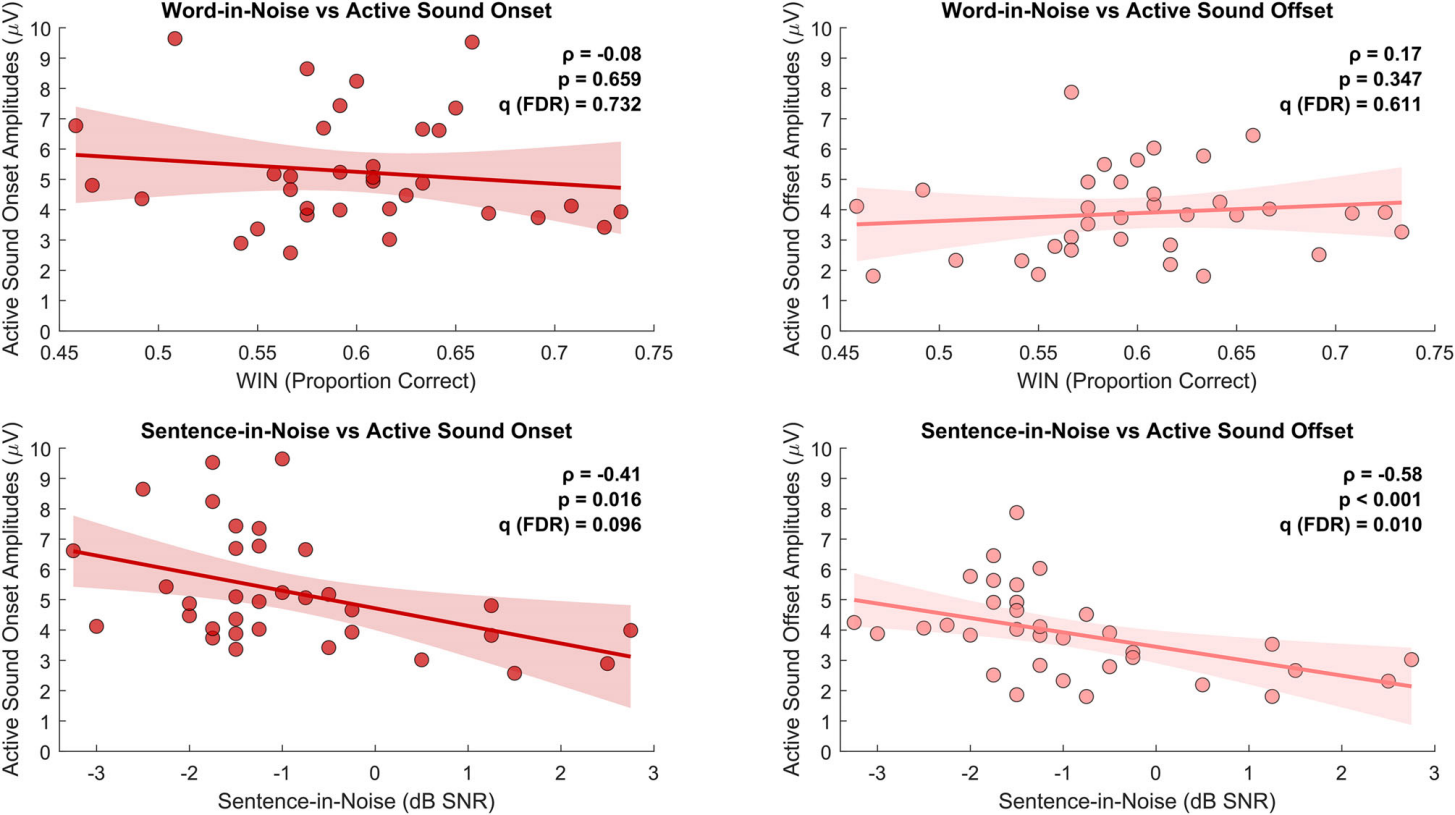
Scatterplots showing correlations between active listening sound‐onset and offset amplitudes and speech‐in‐noise tests The lines of best fit are plotted as straight lines, with shaded areas indicating the 95% confidence intervals for each correlation. The top row presents the relationship between word‐in‐noise (WIN) test scores and active onset amplitudes (left) and active offset amplitudes (right). Neither sound‐onset nor offset amplitudes showed significant correlations with WIN performance (ρ = −0.08, *P* = 0.659, *q* = 0.732; ρ = 0.17, *P* = 0.347, *q* = 0.611, respectively). The bottom row shows the relationship between sentence‐in‐noise test scores and active onset amplitudes (left) and active offset amplitudes (right). A significant association was found between sentence‐in‐noise performance and both sound‐onset (ρ = −0.41, *P* = 0.016, *q* = 0.096) and offset responses (ρ = −0.58, *P* < 0.001, *q* = 0.010). However after applying the FDR correction for multiple comparisons only the relationship between sound‐offset responses and sentence‐in‐noise performance survived.

A further partial correlation analysis was performed to ensure that the relationship between sound‐offset responses in active listening and sentence‐in‐noise performance was not driven by sound‐onset responses, PTA or age. The association between active sound‐offset responses and sentence‐in‐noise performance remained significant after controlling for sound‐onset responses (partial *r* = −0.38, *P* = 0.030), PTA (partial *r* = −0.47, *P* = 0.006) and age (partial *r* = −0.47, *P* = 0.006).

## Discussion

The present study demonstrated that sound‐offset responses can be robustly identified at the single‐subject level in older participants using EEG. Our findings reveal that offset responses are task‐dependent, with the active listening condition eliciting significantly stronger sound‐offset responses. In contrast onset responses were reduced in the active listening condition compared to passive listening, indicating that they did not follow the same task‐related pattern as offset responses. Moreover sound‐offset responses were found to correlate with speech‐in‐noise performance at the sentence level, but not at the word level, even after controlling for pure‐tone hearing thresholds, age and onset responses. The present study, therefore, provides evidence that central auditory sensitivity to sound offsets could be a neural mechanism that underlies speech‐in‐noise difficulties commonly observed in the ageing population.

Previous studies have recorded sound‐offset responses to brief tonal sounds and broadband noise at the cortical level (Baltzell & Billings, 2014; Harris et al., 2012; Horváth et al., 2017; Morse & Vander Werff, 2023). However their experimental designs may not be ideal, as some studies used silent gaps to elicit offset responses, where the short temporal gap between the sound offset and the subsequent onset might create interference with onset responses. Furthermore tonal sounds have limited ecological validity, as they are less representative of real‐world listening conditions. In this study we addressed these issues using 1–2 s white‐noise stimuli in a duration discrimination task to isolate sound‐offset responses and eliminate temporal interactions with sound onsets. All participants demonstrated robust and clearly identifiable offset responses.

The present work provides evidence for task‐dependent modulation in temporal processing, demonstrating that offset responses were significantly larger in the active listening condition compared to the passive condition, whereas onset responses were significantly smaller in the active listening condition than in the passive condition. This finding is partly consistent with results of previous work by Ali et al. (2025), which reported increased offset responses but no task‐related change in onset responses. The smaller onset responses observed in the active task in our study were indeed unexpected. One plausible explanation relates to our design, in which participants always completed the passive EEG condition first followed by the active task. Reduced onset responses in the active condition may therefore reflect stimulus adaptation triggered by repeated sound presentation. The increase in offset responses during active listening suggests a potential attention mechanism involved in sound‐offset encoding, where a top‐down (e.g. endogenous) process also becomes important. More specifically following sound‐onset attentional load, particularly sustained attention, may increase over the course of the stimulus in preparation for detecting the sound offset, as accurate offset detection is critical for estimating sound duration. This increased attentional demand towards the sound offset may contribute to the amplified offset responses in the active listening condition.

The aforementioned reduced onset responses in active listening due to adaptation mechanisms are a valid explanation that might drive this effect, as adaptation in the auditory cortex occurs over time scales ranging from milliseconds to tens of minutes (Fitzgerald & Todd, 2018; Gill & Francis, 2023; Nelken, 2014; Ulanovsky et al., 2004). Interestingly the behaving‐animal literature has reported a similar phenomenon, in which studies have shown suppressed auditory cortex activity during active listening (Otazu et al., 2009; Town et al., 2018). Dong et al. (2013) suggested that neurons in the primary auditory cortex show dual responses, with some suppressed and others enhanced by task engagement. Otazu et al. (2009) proposed that this suppression reflects an early stage of selective routing, reducing activity in neurons irrelevant to the task and thereby limiting the spread of auditory signals to other cortical and subcortical regions. In passive conditions where no task demands are defined auditory activity may instead propagate more broadly, enhancing the overall response to sound stimulation. The pattern observed in animal studies may resemble our human findings to some extent and could be one of the factors driving the smaller onset responses observed during active listening.

There is also evidence that attention can modulate change‐detection responses (Auksztulewicz & Friston, 2015). Sound‐offset responses can be considered a form of change detection, as they represent the transition from sound to silence. It is possible that attention‐modulated responses in offset processing share a common origin with change‐detection mechanisms. In line with this idea Awwad et al. (2023) reported omission responses resembling offset responses in unanaesthetised, but not in anesthetized, rats. However this explanation seems less likely in the current study, as a similar association would be expected for sound‐onset responses, which also involve a transition from silence to sound. Current study, however, does not support an attentional effect on sound‐onset responses in the expected way. One explanation is that onset responses may be evolutionarily more critical, as detecting the onset of a sound (e.g. the rustling of leaves) could signal the presence of a predator or other threats. Therefore onset responses may already be maxed out, leaving little room for further enhancement by attention compared to offset responses. Consistent with this onset responses may be driven by faster and more‐prominent bottom‐up (e.g. exogenous) pathways that prioritize sudden changes in the auditory scene, unlike attention, which involves slower top‐down processing. In contrast to onsets offsets may be less constrained by evolutionary pressures and therefore more susceptible to such top‐down modulation, which could explain the amplified offset responses observed in the active condition. However our findings do not entirely rule out a role for change‐detection mechanisms in offset responses. Larger offset responses observed with increasing stimulus duration in previous studies (Hillyard & Picton, 1978; Song et al., 2024) may reflect greater involvement of change‐detection processes, as such responses scale with the magnitude of the stimulus change (Kim, 2015), for example when a long sound suddenly terminates.

An alternative, more speculative explanation for the smaller onset amplitudes in the active condition could involve an asymmetric functional interdependence between onset and offset responses. Hillyard and Picton (1978) reported a similar inverted relationship, where manipulations that increased offset responses were accompanied by reduced onset responses. At the neural level although onset‐ and offset‐sensitive populations are partly distinct, some auditory cortical neurons can respond to both onset and offset cues, albeit through different sets of synapses (Scholl et al., 2010; Volkov & Galazjuk, 1991). When offset encoding demands are higher these dual‐responsive populations may preferentially contribute to the offset response, which could, in turn, diminish their contribution to the onset response at the scalp level. One possible mechanism is neural refractory effects, where recent offset‐related activity transiently limits the ability of these neurons to generate a strong onset response. Another possibility is a redistribution of processing resources, in which attentional mechanisms bias neural activity towards the task‐relevant offset cue at the expense of onset encoding. In this sense greater offset responses may occur at the expense of smaller onset responses, reflecting a trade‐off rather than an absolute absence of attentional modulation of onset encoding. Further research is required to test whether offset responses can indeed increase at the cost of onset responses and, if so, to identify the mechanisms underlying such a one‐way trade‐off.

A further question concerns sound‐offset responses and their potential clinical relevance in speech‐in‐noise listening. In this study we investigated both sentence‐level and word‐level speech‐in‐noise abilities. We found a significant, large‐sized correlation between sentence‐in‐noise performance and offset responses, but no such correlation for the word‐in‐noise test. One possible explanation is that sound‐offset responses may be more relevant to the glimpsing model of speech‐in‐noise perception. In fluctuating background noise, such as the multitalker babble used in this study, brief silent gaps occur in which the target speech becomes more audible to the listener due to a more favourable signal‐to‐noise ratio (Cooke, 2006). Listening during these dips allows listeners to ‘glimpse’ portions of the target speech (Li & Loizou, 2007). It is likely that we rely more on sound‐offset cues to process these brief silence periods, allowing the target speech to be better glimpsed. However this still does not explain the lack of a relationship between word‐in‐noise performance and sound‐offset responses in this study, as speech glimpsing may facilitate both phoneme and word recognition (Fogerty et al., 2021). The type of word‐in‐noise test used here might underestimate the role of glimpsing in word recognition. Specifically we used a test focused on consonant perception, where only a small portion of the word determines whether the word is correctly recognized. In such cases the probability of glimpsing that exact segment may be lower than in words where glimpsing is critical across the entire word duration. Further research is required to elucidate the role of sound offset in the glimpsing model of speech‐in‐noise listening.

That being said a significant association between word‐in‐noise performance and sound‐offset responses could still be expected, as Kopp‐Scheinpflug et al. (2018) suggested a potential sound‐offset mechanism involved in consonant identification. A study by Hillyard and Picton (1978), as well as an animal study by Solyga and Barkat (2021), showed that cortical sound‐offset amplitudes increase as the duration of the sound increases. This raises the possibility that randomly varying sound duration from 1 to 2 s in our task may not be representative of the brief durations of words in the word‐in‐noise test. Rather it might reinforce the explanation of why sound‐offset responses are correlated with sentence‐in‐noise performance, as the sound durations in our task are more similar to sentence durations than to single words. Considering that sentences are more ecologically valid and more representative of real‐world listening than single words (Geller et al., 2021) we propose that sound‐offset responses could represent a neural substrate underlying real‐world listening difficulties in the population.

Speech‐in‐noise listening is a complex ability that requires multiple auditory cognitive mechanisms. Inhibitory function has been suggested among these mechanisms, where distractions from competing speakers must be inhibited to understand the target speaker (Campbell et al., 2020; Dryden et al., 2017; Mattys et al., 2009). It is well established that inhibitory functions decline with ageing, and that speech‐in‐noise difficulties are a common complaint among older adults (Caspary et al., 2008; Helfer et al., 2017; Smith et al., 2024; Stothart & Kazanina, 2016). The role of inhibitory function at the neural level in the central auditory system is less understood. Healthy central auditory processing relies on a delicate balance between excitatory and inhibitory neural mechanisms. A recent study by Harris et al. (2022) demonstrated that decreased inhibition may lead to increased central gain and poorer speech‐in‐noise performance in older adults. Sound‐offset responses have been proposed as a potential tool to monitor inhibitory function in the auditory pathway, as the sound‐offset pathway in the auditory brain includes GABA, an inhibitory neurotransmitter (Morse & Vander Werff, 2023). Furthermore Dobri and Ross (2021) reported that total GABA levels in the auditory cortex are correlated with speech‐in‐noise performance in older adults. In this study the significant association observed between sentence‐in‐noise performance and offset responses may be driven by inhibitory function. More speculatively greater offset responses in older adults may indicate less‐impaired inhibitory function and, thus, a healthier central auditory system. We argue that offset responses could explain some portion of the speech‐in‐noise deficits observed in older adults.

This study has a few limitations. First the number of trials in the active listening condition varied across participants. This is because we determined the thresholds based on the number of reversals. Because individuals may show varying levels of performance this could influence onset and offset responses, as trials that are far from the threshold level might be less cognitively demanding and result in smaller onset and offset responses. On the contrary using a reversal‐based threshold calculation allowed us to ensure that all participants experienced a similarly demanding cognitive process. Second the correlational analyses were exploratory and conducted in a relatively small sample, making the results susceptible to both inflated effect sizes and reduced power to detect true effects, as the case in all studies using small sample sizes (Button et al., 2013; Yarkoni, 2009). Although we controlled for multiple comparisons using the Benjamini–Hochberg procedure, the possibility of false positives remains, and these findings should be interpreted with caution. Larger studies are needed to better establish the role of sound‐offset encoding in speech‐in‐noise perception and to clarify the underlying mechanisms. Finally although this study primarily focused on older adults due to their well‐known listening difficulties, the potential relationship between speech‐in‐noise performance and offset responses should be replicated in other age groups. Future studies should also explore the clinical applications of sound‐offset responses, particularly for individuals with listening difficulties. This is especially relevant for cochlear implant and hearing aid users, who often struggle to understand speech in real‐world listening environments, as both devices currently lack efficient processing of abrupt changes in sound, such as onsets and offsets.

### Conclusion

This work provides compelling evidence that sound‐offset responses can be robustly identified at the single‐subject level using EEG, which might enable future clinical applications. Our findings highlight that offset responses are task‐dependent, showing a significant increase under active listening, whereas onset responses were unexpectedly reduced compared to passive listening. Importantly sound‐offset responses were also associated with speech‐in‐noise ability at the sentence level. This suggests that weak sound‐offset encoding may be an underlying factor in listening difficulties, and that offset responses may reflect a neural correlate of speech‐in‐noise perception.

## Additional information

## Competing interests

The authors declare no competing interests.

## Author contributions

H.C. conceptualised and designed the study, collected data, performed data analysis and interpretation and wrote the manuscript. X.G. and E.B. conceptualised and designed the study and performed data analysis. A.S., W.S. and J.F.L. conceptualised and designed the study and performed data interpretation. T.D.G. conceptualised and designed the study, performed data interpretation and secured funding. All authors have approved the final version of the manuscript and agreed to be accountable for all aspects of the work. All persons designated as authors qualify for authorship, and all those who qualify for authorship are listed.

## Funding

This work was supported by the MRC (grant number 815 MR/T032553/1). Contributions of J.F.L. and A.S. were supported by the NIHR‐UCL Biomedical Research Centre Hearing Health Theme.

## Supporting information




Peer Review History

Supplementary Material

## Data Availability

The data that support the findings of this study are available from https://osf.io/skd93/?view_only=b166226fdff64bb89a8cc163e403f5a0.
