# Supplementary material for: Sound‐offset encoding is related to speech‐in‐noise perception at sentence level in older adults
Source: J Physiol. 2025 Dec 16;604(21):8767–80. doi: 10.1113/JP289104 (PMC13725239; doi:10.1113/JP289104)
Supplement: Supplementary file 1 [file TJP-604-8767-s002.pdf]

The Journal of Physiology

Sound Offset Encoding is Related to Speech-in-Noise Perception at Sentence Level in Older Adults

Hasan Colak, Xiaoxuan Guo, Adèle Simon, Ester Benzaquén, William Sedley, Jennifer F. Linden, and Timothy D. Griffiths
DOI: 10.1113/JP289104

Corresponding author(s): Hasan Colak (h.colak2@newcastle.ac.uk)

The following individual(s) involved in review of this submission have agreed to reveal their identity: Katrin Krumbholz (Referee #2)

Review Timeline:

Submission Date:	16-Apr-2025
Editorial Decision:	24-Jul-2025
Revision Received:	14-Sep-2025
Editorial Decision:	20-Oct-2025
Revision Received:	11-Nov-2025
Editorial Decision:	26-Nov-2025
Revision Received:	28-Nov-2025
Accepted:	02-Dec-2025

Senior Editor: Vaughan Macefield

Reviewing Editor: Conny Kopp-Scheinpflug

Transaction Report:

(Note: With the exception of the correction of typographical or spelling errors that could be a source of ambiguity, letters and reports are not edited. Depending on transfer agreements, referee reports obtained elsewhere may or may not be included in this compilation. Referee reports are anonymous unless the Referee chooses to sign their reports.)

1st Editorial Decision 24-Jul-2025

Dear Dr Colak,

Re: JP-RP-2025-289104 "**Sound Offset Encoding is Related to Speech-in-Noise Perception at Sentence Level in Older Adults**" by Hasan Colak, Xiaoxuan Guo, Adèle Simon, Ester Benzaquén, William Sedley, Jennifer F. Linden, and Timothy D. Griffiths

Thank you for submitting your manuscript to The Journal of Physiology. It has been assessed by a Reviewing Editor and by 3 expert referees and we are pleased to tell you that it is potentially acceptable for publication following satisfactory major revision.

Please advise your co-authors of this decision as soon as possible.

The referee reports are copied at the end of this email.

Please address all the points raised and incorporate all requested revisions or explain in your Response to Referees why a change has not been made. We hope you will find the comments helpful and that you will be able to return your revised manuscript within 2 months. If you require longer than this, please contact journal staff: jp@physoc.org. Please note that this letter does not constitute a guarantee for acceptance of your revised manuscript.

Your revised manuscript should be submitted online using the link in your Author Tasks: Link Not Available. This link is accessible via your account as Corresponding Author; it is not available to your co-authors. If this presents a problem, please contact journal staff (jp@physoc.org). Image files from the previous version are retained on the system. Please ensure you replace or remove any files that are being revised.

If you do not wish to submit a revised version of your manuscript, you must inform our journal staff (jp@physoc.org) or reply to this email to request withdrawal. Please note that a manuscript must be formally withdrawn from the peer review process at one journal before it may be submitted to another journal.

TRANSPARENT PEER REVIEW POLICY: To improve the transparency of its peer review process, The Journal of Physiology publishes online as supporting information the peer review history of all articles accepted for publication. Readers will have access to decision letters, including Editors' comments and referee reports, for each version of the manuscript, as well as any author responses to peer review comments. Referees can decide whether or not they wish to be named on the peer review history document.

ABSTRACT FIGURES: Authors are expected to use The Journal's premium BioRender account to create/redraw their Abstract Figures. Information on how to access this account is here: https://physoc.onlinelibrary.wiley.com/journal/14697793/biorender-access.

This will enable Authors to create and download high-resolution figures. If authors have used the free BioRender service, they can use the instructions provided in the link above to download a high-resolution version suitable for publication.

The link provided should only be used for the purposes of this submission. Authors will be charged for figures created on this account if they are not related to this manuscript submission.

LANGUAGE EDITING AND SUPPORT FOR PUBLICATION: If you would like help with English language editing, or other article preparation support, Wiley Editing Services offers expert help, including English Language Editing, as well as translation, manuscript formatting, and figure formatting at www.wileyauthors.com/eeo/preparation. You can also find resources for Preparing Your Article for general guidance about writing and preparing your manuscript at www.wileyauthors.com/eeo/prepresources.

RIGOUR AND REPRODUCIBILITY: We recommend authors consult The Journal's Rigour and Reproducibility requirements to ensure that their manuscript is suitable for publication: https://physoc.onlinelibrary.wiley.com/pb-assets/hub-assets/physoc/documents/TJP-Rigour-and-Reproducibility-Requirements.pdf

REVISION CHECKLIST:

Check that your Methods section conforms to journal policy: https://jp.msubmit.net/cgi-bin/main.plex?form_type=display_requirements#methods.

Check that data presented conforms to the statistics policy: https://jp.msubmit.net/cgi-bin/main.plex?form_type=display_requirements#statistics.

Upload a full Response to Referees file. To create your 'Response to Referees': copy all the reports, including any comments from the Senior and Reviewing Editors, into a Microsoft Word, or similar, file and respond to each point, using font or background colour to distinguish comments and responses and upload as the required file type.

Please upload two versions of your manuscript text: one with all relevant changes highlighted and one clean version with no changes tracked. The manuscript file should include all tables and figure legends, but each figure/graph should be uploaded as separate, high-resolution files.

You may also upload:

- 'Potential Cover Art' for consideration as the issue's cover image
- Appropriate Supporting Information (Video, audio or data set: see https://jp.msubmit.net/cgi-bin/main.plex?form_type=display_requirements#supp).

We look forward to receiving your revised submission.

If you have any queries, please reply to this email and we will be pleased to advise.

Yours sincerely,

Vaughan Macefield
Senior Editor
The Journal of Physiology

REQUIRED ITEMS

- Author photo and profile. First or joint first authors are asked to provide a short biography (no more than 100 words for one author or 150 words in total for joint first authors) and a portrait photograph. These should be uploaded and clearly labelled together in a Word document with the revised version of the manuscript. See Information for Authors for further details.

- You must start the Methods section with a paragraph headed Ethical Approval. If experiments were conducted on humans, confirmation that informed consent was obtained, preferably in writing, that the studies conformed to the standards set by the latest revision of the Declaration of Helsinki and that the procedures were approved by a properly constituted ethics committee, which should be named, must be included in the article file. If the research study was registered (clause 35 of the Declaration of Helsinki), the registration database should be indicated, otherwise the lack of registration should be noted as an exception (e.g. The study conformed to the standards set by the Declaration of Helsinki, except for registration in a database). For further information see: https://physoc.onlinelibrary.wiley.com/hub/human-experiments.

- Your manuscript must include a complete Additional Information section, including competing interests; funding; author contributions and acknowledgements.

EDITOR COMMENTS

Reviewing Editor:

Dear authors, your manuscript has been reviewed by 3 experts in the field. While they do point out major weaknesses in the current manuscript regarding details of data analysis, the statistical tests used and the interpretation of the data; reviewer 3 nicely sums up the importance of your manuscript: "that offset responses can be robustly measured, are task-sensitive, and show some correlation with behavioral performance, in particular sentence in noise performance". Given the imbalance of knowledge on onset responses vs that of offset responses, I would like to urge you to step-by-step improve the manuscript based on the reviewers' suggestions, so that the information on the offset responses can eventually be published and help to close this gap in knowledge.

Please be aware that this is indeed a major revision, which under previous policies may have qualified for a soft rejection with permission to re-submit. That said - please let us know if you need more time to do a detailed revision.

Please also see 'Required Items' above.

Senior Editor:

Thank you for submitting your manuscript to The Journal of Physiology. We have received three sets of assessments from independent reviewers. As you will see below, the Reviewing Editor is offering you the opportunity to submit a fully revised manuscript that requires detailed responses to all comments before we can consider this further, a view with which I concur. Of course, we cannot guarantee eventual acceptance of the manuscript but trust you will be willing to submit a revised version.

REFEREE COMMENTS

Referee #1:

This study investigates how the brain's ability to encode sound offsets (i.e., when a sound ends) contributes to older adults' ability to understand speech in noisy environments. Thirty-four participants aged 60-81 underwent hearing tests, speech-in-noise assessments, and EEG recordings during active and passive listening tasks. The key finding is that sound-offset responses were reliably detected at the individual level using EEG and that these responses were significantly larger during active listening (when participants focused on sounds) than during passive listening. In contrast, sound-onset responses did not vary between conditions, suggesting offset responses are more influenced by attention. The authors provide a range of correlation analyses and further identify that stronger active sound-offset responses correlated with better sentence-level speech-in-noise perception, even after controlling for age and hearing loss. No such correlation was found with word-level speech tests or other parameters tested. The authors conclude that EEG measures of offset responses could serve as a clinical biomarker for diagnosing and understanding listening difficulties in older adults and that sound-offset processing may play a key role in understanding speech amidst background noise, especially at the sentence level.

I appreciate the smart study design that facilitate both the subjects focusing on sound offset as well as reliably identification of offset signals in the EEG recordings. The data analysis is thorough and the results interesting and mostly convincing. I have only a few questions and remarks.

1. The authors suggest that sound-offset responses, but not onset responses, are modulated by attention, as reflected by increased EEG amplitudes in active versus passive listening. However, in the duration discrimination paradigm used, accurate judgment of stimulus length inherently relies on both sound onset and offset cues-especially given the randomization of inter-stimulus intervals. This raises the question how the authors reconcile the task-relevance of onset cues with the apparent absence of attentional modulation in onset responses?

2. Somewhat related to the former comment, in Figure 4A, the group-level comparison suggests no significant difference in onset amplitude between active and passive listening. However, the corresponding scatter plot shows a clear difference for individuals to exhibit reduced amplitudes during active listening. Did the authors conduct a paired statistical analysis on individual onset amplitude changes? This approach may yield more nuanced insights into within-subject effects.

3. Given the study's hypothesis that enhanced offset processing supports temporal discrimination and speech-in-noise comprehension, one could anticipate performance in the Duration Discrimination Task (DDT) to correlate with attentional modulation of offset EEG amplitudes. I.e., individuals with better DDT thresholds could show a larger increase in offset amplitude from passive to active conditions. Did the authors analyze the relationship between DDT performance and the differential offset response across listening conditions? Such a finding would directly support the attentional sensitivity of the offset pathway and its behavioral relevance.

5. Line 163: update placeholder by correct file number.

Referee #2:

Review: "Sound offset encoding is related to speech-in-noise perception at sentence level in older adults" by Hasan Colak et al.

This study describes EEG measurements of auditory-evoked ON- and OFF-responses in a group of 34 older adults (6-81 years of age) with near-normal hearing. The responses were elicited by 1-2-s, 75-dB SPL noise bursts, separated by 1-2 s silent gaps, and were recorded in both active and passive listening conditions. The aim was to test whether OFF-responses could be measured at a single-subject level and whether their size, measured between the FCz and combined P9/10 channels, is significantly associated with gap detection, duration discrimination, or speech-in-noise (SiN) reception

performance. The results showed (i) significant OFF-responses in every subject; (ii) attentional effects (difference between active and passive recording conditions) in the OFF- but not ON-response; (iii), significant positive association between active OFF-response size and sentence-, but not word-in-noise performance. Based on these results, the authors suggest that the OFF-response may be a "potential clinical measure of auditory cognition relevant to speech-in-noise listening".

This study has several major shortcomings, which mean that the overall knowledge advance created is likely minimal and, in fact, the study results may be inadvertently misleading. A major problem is that it is not clear how exactly the study was motivated; what questions was it intended to answer, or what hypotheses was it designed to test?

The auditory-evoked OFF-response has been subject to extensive research, much of which took place in the previous century. This has shown that the occurrence of an OFF-response depends crucially on the eliciting sound duration (e.g., Hillyard & Picton, 1978, "ON and OFF components in the AEP"; Song et al., 2024, "A new function of offset response in the primate auditory cortex: marker of temporal integration"), and that, when the eliciting sound is more than a few 100 ms long, an OFF-response generally occurs just as reliably as the ON-response. Therefore, the first finding of "robust [...] offset responses at a single-subject level" is not in any way novel or surprising.

The second finding, that the OFF-response was larger in the active than passive listening condition, whereas the ON-response was statistically unchanged, conflicts with an extensive body of previous research showing that the ON-response can be subject to substantial attentional modulation (typically, the ON-response approximately doubles in size when attention is actively focussed on the eliciting sound). This finding is thus specific to the current experimental design carries no general validity or value. Looking at the current data, it is interesting to note that the ON-response appeared to be slightly, but largely consistently, smaller in the active compared to passive listening condition. A similar inverted relationship of the ON-response size was also observed by Hillyard & Picton (1978) in a study that varied the OFF-response size by varying sound duration, perhaps indicating some form of asymmetric functional interdependence between the two types of responses (whereby a larger OFF-response causes a smaller ON-response but not vice versa).

The remaining findings are purely correlational, which, without a clearly stated prior aim or question, and given the relatively small sample size, is highly problematic, particularly, because the authors only discuss findings that survive their relatively stringent family-wise error threshold (Bonferroni correction for 45 comparisons; where exactly does this number come from?). I presume that the authors' expectation of a correlation with SiN performance was predicated on the idea that the OFF-response may play a functional role in the identification of stop consonants, where the initial burst and subsequent release or voicing onset are separated by a short silent gap, the duration of which determines the perceived consonant category (as hypothesized, e.g., by Kopp-Scheinpflug et al., 2018). Whilst Dr Kopp-Scheinpflug's argument may be valid at the microscopic (single-neuron) level, it almost certainly isn't valid at the level of the gross, long-latency OFF-responses measured with EEG and MEG. This is because, as I pointed out above, the occurrence of these OFF-responses depends on the preceding sound duration, with an OFF-response occurring only when the preceding sound is several 100's of ms long. Therefore, OFF-responses can't be functionally essential for stop-consonant identification, which human listeners can happily accomplish even when consonants are presented in isolation, and thus the silent gap is preceded by only a very brief burst. For the reader, it thus remains unclear as to why a correlation between the OFF-response size and SiN performance should be expected and, assuming the observed correlation is true, how such correlation may be brought about. On the other hand, the more obvious correlations of OFF-response size with gap detection and duration discrimination performance, which the authors almost certainly expected to find at the outset of their study, are barely mentioned at all, merely because they don't survive the family-wise error criterion. This epitomizes the power problem in small correlational studies with multiple comparisons (Yarkoni, 2009, "Big correlations in little studies"; see also Ioannidis, 2005, "Why most published research findings are false"; and Button et al., 2013, "Power failure: why small sample size undermines the reliability of neuroscience"): To guard against family-wise type-I error, such studies set perniciously high type-I error thresholds (of $p < 0.05/M$, where M is the number of comparisons conducted). As a result, the probability of detecting a 'true' effect (i.e., an effect that is actually present) in each single comparison becomes very small, even if the actual/true effect size is comparatively large, thus leading to an inflated type-II error rate (i.e., likelihood of missing a true effect), whilst at the same time inflating the observed effect size in those comparisons that manage, by chance, to surpass the significance threshold.

As a result of the latter effect of low power (i.e., overestimation of the effect size of significant effects), the idea that the OFF-response may have potential as a clinical marker for speech-relevant cognitive functions is widely over-optimistic. The current data suggest that the OFF-response size in a single EEG electrode (PCz vs P9/10) explains as much as one third (33%) of inter-individual variance in sentence-in-noise perception in older, albeit near-normal hearing listeners. Given the vast degree of variance in both SiN performance and long-latency cortical auditory-evoked potentials, particularly when measured within individual electrodes (i.e., not taking into account variation in response scalp distribution), I would expect

the real effect (if there is one) to be more of the order 1% or even less.

Given the relatively low-level (automatic) nature of the N1-P2, the main components of the OFF-response, I would also assume that any true association between OFF-response size and SiN performance is artefactual, or trivial, in nature, likely caused by variation in low-frequency hearing thresholds (subclinical in the current study). Calculating the partial correlation between OFF-response size and SiN performance with respect to average hearing thresholds (the so-called PTA, which the current authors calculate across the entire audiometric range from 0.25 to 8 kHz) does not necessarily mean that a potential confounding effect of hearing thresholds has been successfully precluded. This is because, firstly, the effect of hearing thresholds on OFF-response size, SiN performance or both may be non-linear and thus inadequately accounted for by linear partialling out. More importantly, averaging hearing thresholds to a single, wideband PTA measure may have diluted the effects of low-frequency thresholds, which, even though the stimulus was a wideband noise, would be expected to have the greatest effects on long-latency cortical auditory-evoked responses, such as the OFF-response (this is due to the layout of the cortical tonotopic map, which favours low-frequency response contributions).

Overall, I feel that the authors should focus much more on what they originally set out to achieve, rather than moulding the story to suit the 'significant' results.

Referee #3:

The work presented here measured onset and offset responses in older listeners, and related those to a number of behavioral performance measures: duration discrimination, gap detection, word in noise and sentence in noise. The most important result (in my eyes) is that offset responses can be robustly measured, are task-sensitive (larger in active than in passive listening), and show some correlation with behavioral performance, in particular sentence in noise performance.

However, there are significant weaknesses in the data analysis and the way conclusions are derived from the data. The most important is that repeated assertion that there was no task dependence in the onset responses. This claim is directly falsified by the data in Fig. 4B. I would guess that the tests showing lack of significant difference were unpaired. However, the data is paired (active and passive P2-N1 measured in the same subject), and across-subject variability is considerable (as obvious from Fig both panels of Fig. 4), artificially reducing the significance of unpaired tests. A paired comparison between the active and passive onset P2-N1 values give a strongly significant difference (even the weak sign test, which I could perform with the data in Fig. 4B, gives a highly significant result). Since this is a crucial part of the claims in this paper, this manuscript as it stands is wrong.

While I commend the authors for supplying what seems to be their full dataset, this is insufficient for checking the correctness of the analysis - they need to supply also the analysis routines they used, and even better, the scripts used to generate the figures and run the statistics. Furthermore, their dataset lacks any documentation, making it effectively useless.

Major comments:

The introduction doesn't make sense in the context of the paper. The main result is the different effects of active vs. passive listening on the offset responses, but the effects of attention on offset responses is not discussed at all, or the robustness and potential source populations for offset-dependent ERP components is not discussed. Instead, the intro speaks mostly about the importance of offset responses to speech understanding, which is not the only or even main point of the paper.

The references in the introduction are very partial - important animal work is missing (e.g. the work of the group of Mike Wehr).

Analysis of Table 2: The Bonferroni correction here is based on 45 comparisons. However, only a much smaller number of correlation coefficients here are really interesting. The matrix correlates everything with everything, but the interesting correlations are only those that pertain to the behavioral measures vs. electrical measures - these are the crucial tests of this paper. There are 5 of each, giving in total only 25 interesting comparisons. More importantly, these values are highly correlated among themselves, and certainly don't fit the assumption of the Bonferroni correction (independence of the tests that are being conducted). Thus, this approach doesn't make sense.

There are other correction schemes (e.g. Benjamini-Hochberg) and other approaches to the study of correlation structure (e.g. factor analysis and related methodologies) that make much more sense here.

I'm a bit surprised that the gap detection performance showed such poor correlation with the speech in noise tests. Some clinical literature suggests that gap detection should correlated with speech performance.

Minor comments:

Discussion: around lines 380, critique of the ecological validity of pure tones: I don't think that 1-s lone white noise is more ecological than pure tones. This is not a serious argument and I would remove it.

Lines ~400, offset responses and change detection: Awwad et al. (2021) showed that offset responses, as well as some sort of omission responses, occur in awake but not anesthetized rats. Maybe worth mentioning here, this has the same flavor.

Line 163: the ethics committee name is corrupted.

Line 289 refers to supplementary material 1, I couldn't locate it.

END OF COMMENTS

EDITOR COMMENTS

Reviewing Editor:

Dear authors, your manuscript has been reviewed by 3 experts in the field. While they do point out major weaknesses in the current manuscript regarding details of data analysis, the statistical tests used and the interpretation of the data; reviewer 3 nicely sums up the importance of your manuscript: "that offset responses can be robustly measured, are task-sensitive, and show some correlation with behavioral performance, in particular sentence in noise performance". Given the imbalance of knowledge on onset responses vs that of offset responses, I would like to urge you to step-by-step improve the manuscript based on the reviewers' suggestions, so that the information on the offset responses can eventually be published and help to close this gap in knowledge.

Please be aware that this is indeed a major revision, which under previous policies may have qualified for a soft rejection with permission to re-submit. That said - please let us know if you need more time to do a detailed revision.

Please also see 'Required Items' above.

RESPONSE: Dear Editor, we would like to thank you for taking the time to review our paper. We are pleased to submit a significantly revised version of our manuscript based on the comments from the three reviewers. We believe this research will help bridge the gap in knowledge on sound-offset processing.

Senior Editor:

Thank you for submitting your manuscript to The Journal of Physiology. We have received three sets of assessments from independent reviewers. As you will see below, the Reviewing Editor is offering you the opportunity to submit a fully revised manuscript that requires detailed responses to all comments before we can consider this further, a view with which I concur. Of course, we cannot guarantee eventual acceptance of the manuscript but trust you will be willing to submit a revised version.

RESPONSE: Dear Editor, we would like to thank you for taking the time to review our paper and for considering it for publication in the Journal of Physiology. We are pleased to submit a significantly revised version of our manuscript based on the comments from the three reviewers.

REFEREE COMMENTS

Referee #1:

This study investigates how the brain's ability to encode sound offsets (i.e., when a sound ends) contributes to older adults' ability to understand speech in noisy environments. Thirty-four participants aged 60-81 underwent hearing tests, speech-in-noise assessments, and EEG recordings during active and passive listening tasks. The key finding is that sound-offset responses were reliably detected at the individual level using EEG and that these responses were significantly larger during

active listening (when participants focused on sounds) than during passive listening. In contrast, sound-onset responses did not vary between conditions, suggesting offset responses are more influenced by attention. The authors provide a range of correlation analyses and further identify that stronger active sound-offset responses correlated with better sentence-level speech-in-noise perception, even after controlling for age and hearing loss. No such correlation was found with word-level speech tests or other parameters tested. The authors conclude that EEG measures of offset responses could serve as a clinical biomarker for diagnosing and understanding listening difficulties in older adults and that sound-offset processing may play a key role in understanding speech amidst background noise, especially at the sentence level.

I appreciate the smart study design that facilitate both the subjects focusing on sound offset as well as reliably identification of offset signals in the EEG recordings. The data analysis is thorough and the results interesting and mostly convincing. I have only a few questions and remarks.

1. The authors suggest that sound-offset responses, but not onset responses, are modulated by attention, as reflected by increased EEG amplitudes in active versus passive listening. However, in the duration discrimination paradigm used, accurate judgment of stimulus length inherently relies on both sound onset and offset cues-especially given the randomization of inter-stimulus intervals. This raises the question how the authors reconcile the task-relevance of onset cues with the apparent absence of attentional modulation in onset responses?

RESPONSE: Dear Reviewer,

Thank you for taking the time to read our paper and for your helpful suggestions to improve the study.

We agree that both sound onset and offset encoding are important for duration discrimination performance in the current design, and we appreciate this thoughtful question. Our results suggest that offset responses were more susceptible to attention-related modulation than onset responses. There are several possible explanations for this finding. One possibility is that participants begin allocating attention at stimulus onset, with attentional load gradually increasing and peaking toward the end of the stimulus. It is likely that this attentional load reaches its peak around sound offset, making offset responses more sensitive to task-related changes (see relevant section in the Discussion, quoted below). We also put forward an alternative explanation in the discussion, arguing that sound onset responses might already be maxed out due to evolutionary reasons. It is hard to prove the point with the latter, but these could be potential reasons why we do not see such task-related increases in the onset responses (see the second paragraph below).

“The present work provides evidence for task-dependent modulation in temporal processing, demonstrating that offset responses were significantly larger in the active listening condition compared to the passive condition, whereas onset responses were significantly smaller in the active listening condition than in the passive condition. This finding is partly consistent with results of previous work by Ali et al. (2025), which reported increased offset responses but no task-related change in onset responses. The smaller onset responses observed in the active task in our study were indeed unexpected. One plausible explanation relates to our design, in which participants always completed the passive EEG condition first, followed by the active task. Reduced onset responses in the active condition may therefore reflect stimulus adaptation triggered by repeated sound presentation. The increase in offset responses during active listening suggests a potential attention mechanism involved in sound-offset encoding, where a top-down (e.g., endogenous) process also becomes important. More specifically, following sound onset, attentional load, sustained attention in particular, may increase over the course of the stimulus in preparation for detecting the sound offset,

as accurate offset detection is critical for estimating sound duration. This increased attentional demand toward the sound offset may contribute to the amplified offset responses in the active listening condition."

"There is also evidence that attention can modulate change-detection responses (Auksztulewicz & Friston, 2015). Sound-offset responses can be considered a form of change detection, as they represent the transition from sound to silence. It is possible that attention-modulated responses in offset processing share a common origin with change-detection mechanisms. In line with this idea, Awwad et al. (2023) reported omission responses resembling offset responses in unanaesthetised, but not in anesthetized rats. However, this explanation seems less likely in the current study, as a similar association would be expected for sound-onset responses, which also involve a transition from silence to sound. Current study, however, does not support an attentional effect on sound-onset responses in the expected way. One explanation is that onset responses may be evolutionarily more critical, as detecting the onset of a sound (e.g., the rustling of leaves) could signal the presence of a predator or other threats. Therefore, onset responses may already be maxed out, leaving little room for further enhancement by attention compared to offset responses. Consistent with this, onset responses may be driven by faster and more prominent bottom-up (e.g., exogenous) pathways that prioritize sudden changes in the auditory scene, unlike attention, which involves slower top-down processing. In contrast to onsets, offsets may be less constrained by evolutionary pressures and therefore more susceptible to such top-down modulation, which could explain the amplified offset responses observed in the active condition. However, our findings do not entirely rule out a role for change-detection mechanisms in offset responses. Larger offset responses observed with increasing stimulus duration in previous studies (Hillyard & Picton, 1978; Song et al., 2024) may reflect greater involvement of change-detection processes, since such responses scale with the magnitude of the stimulus change (Kim, 2015), for example when a long sound suddenly terminates."

2. Somewhat related to the former comment, in Figure 4A, the group-level comparison suggests no significant difference in onset amplitude between active and passive listening. However, the corresponding scatter plot shows a clear difference for individuals to exhibit reduced amplitudes during active listening. Did the authors conduct a paired statistical analysis on individual onset amplitude changes? This approach may yield more nuanced insights into within-subject effects.

RESPONSE: Thank you for pointing out this important issue. In the first analysis, we did not pair the data. This unpaired approach may have underestimated the true difference between active and passive listening within the same subjects. In this revision, we have implemented a paired analysis comparing active and passive conditions for both onset and offset responses.

This paired analysis revealed significantly smaller onset amplitudes during the active listening condition compared to the passive condition. This was indeed an unexpected finding. We believe it can be explained by the fixed task order, where all participants completed the passive condition first, followed by the active condition (in this revision, we also corrected an error in our original description of the task order. This can be verified by checking the EEG recording timestamps in the uploaded datasets, see below). To avoid any confusion, we would like to clarify that all participants completed the passive condition first, followed by the active condition. The reduced onset amplitudes in the active condition may reflect adaptation or habituation due to repetitive sound presentation. We have also revised the relevant discussion paragraph, as quoted above:

"The present work provides evidence for task-dependent modulation in temporal processing, demonstrating that offset responses were significantly larger in the active listening condition compared to the passive condition, whereas onset responses were significantly smaller in the active

listening condition than in the passive condition. This finding is partly consistent with results of previous work by Ali et al. (2025), which reported increased offset responses but no task-related change in onset responses. The smaller onset responses observed in the active task in our study were indeed unexpected. One plausible explanation relates to our design, in which participants always completed the passive EEG condition first, followed by the active task. Reduced onset responses in the active condition may therefore reflect stimulus adaptation triggered by repeated sound presentation. The increase in offset responses during active listening suggests a potential attention mechanism involved in sound-offset encoding, where a top-down (e.g., endogenous) process also becomes important. More specifically, following sound onset, attentional load, sustained attention in particular, may increase over the course of the stimulus in preparation for detecting the sound offset, as accurate offset detection is critical for estimating sound duration. This increased attentional demand toward the sound offset may contribute to the amplified offset responses in the active listening condition."

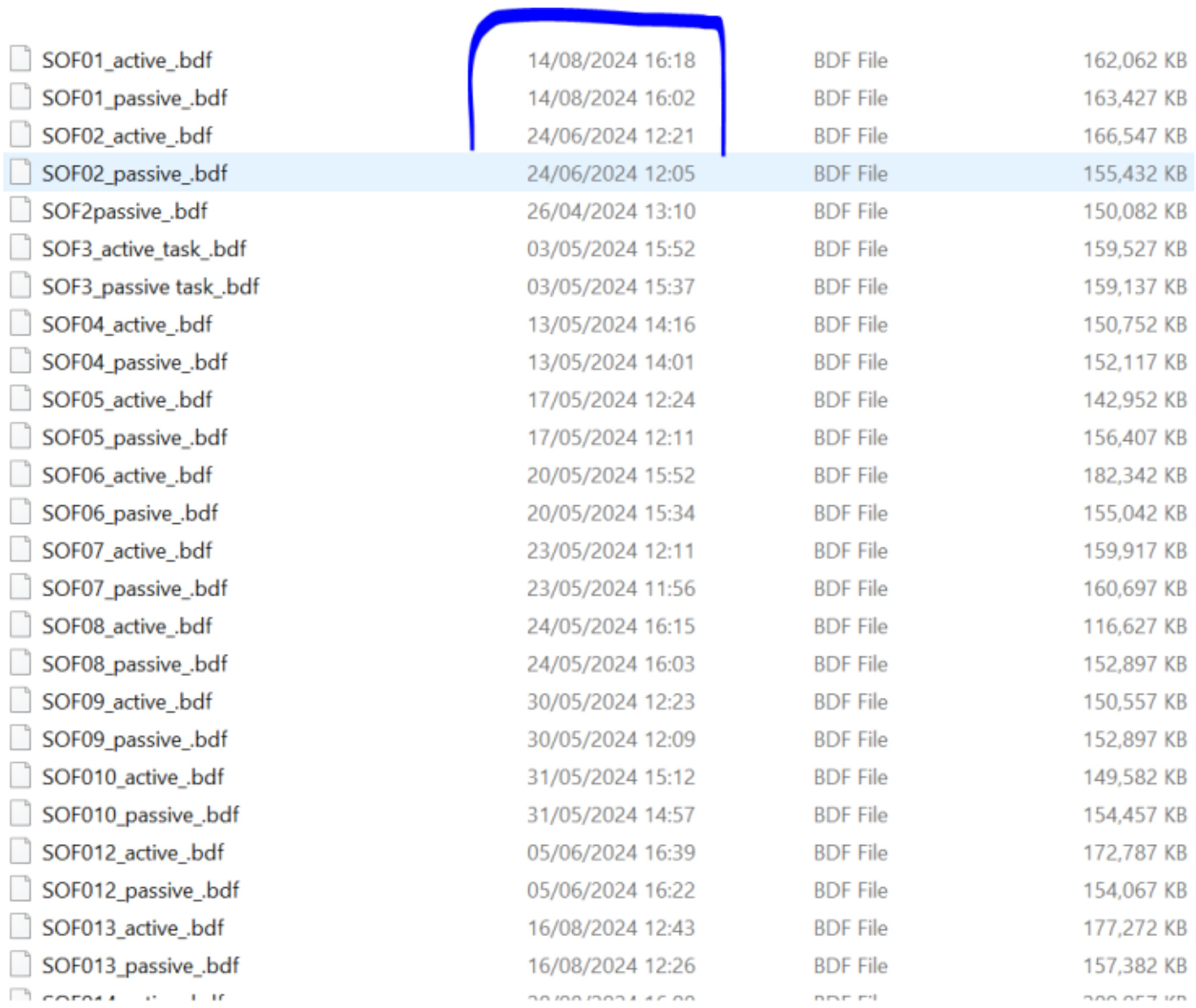

SOF01_active_.bdf	14/08/2024 16:18	BDF File	162,062 KB
SOF01_passive_.bdf	14/08/2024 16:02	BDF File	163,427 KB
SOF02_active_.bdf	24/06/2024 12:21	BDF File	166,547 KB
SOF02_passive_.bdf	24/06/2024 12:05	BDF File	155,432 KB
SOF2passive_.bdf	26/04/2024 13:10	BDF File	150,082 KB
SOF3_active_task_.bdf	03/05/2024 15:52	BDF File	159,527 KB
SOF3_passive task_.bdf	03/05/2024 15:37	BDF File	159,137 KB
SOF04_active_.bdf	13/05/2024 14:16	BDF File	150,752 KB
SOF04_passive_.bdf	13/05/2024 14:01	BDF File	152,117 KB
SOF05_active_.bdf	17/05/2024 12:24	BDF File	142,952 KB
SOF05_passive_.bdf	17/05/2024 12:11	BDF File	156,407 KB
SOF06_active_.bdf	20/05/2024 15:52	BDF File	182,342 KB
SOF06_pasive_.bdf	20/05/2024 15:34	BDF File	155,042 KB
SOF07_active_.bdf	23/05/2024 12:11	BDF File	159,917 KB
SOF07_passive_.bdf	23/05/2024 11:56	BDF File	160,697 KB
SOF08_active_.bdf	24/05/2024 16:15	BDF File	116,627 KB
SOF08_passive_.bdf	24/05/2024 16:03	BDF File	152,897 KB
SOF09_active_.bdf	30/05/2024 12:23	BDF File	150,557 KB
SOF09_passive_.bdf	30/05/2024 12:09	BDF File	152,897 KB
SOF010_active_.bdf	31/05/2024 15:12	BDF File	149,582 KB
SOF010_passive_.bdf	31/05/2024 14:57	BDF File	154,457 KB
SOF012_active_.bdf	05/06/2024 16:39	BDF File	172,787 KB
SOF012_passive_.bdf	05/06/2024 16:22	BDF File	154,067 KB
SOF013_active_.bdf	16/08/2024 12:43	BDF File	177,272 KB
SOF013_passive_.bdf	16/08/2024 12:26	BDF File	157,382 KB

3. Given the study's hypothesis that enhanced offset processing supports temporal discrimination and speech-in-noise comprehension, one could anticipate performance in the Duration Discrimination Task (DDT) to correlate with attentional modulation of offset EEG amplitudes. I.e., individuals with better DDT thresholds could show a larger increase in offset amplitude from passive to active conditions. Did the authors analyze the relationship between DDT performance and the differential offset response across listening conditions? Such a finding would directly support the attentional sensitivity of the offset pathway and its behavioral relevance.

RESPONSE: We are deeply grateful for your suggestion. A potential difference between active and passive offset amplitudes might relate to behavioural duration discrimination performance and could reflect attentional modulation. As you advised, we calculated the ratio of active to passive offset amplitudes and examined its correlation with behavioural duration discrimination thresholds (DDT). Although it is a good hypothesis to test the correlation between behavioural performance and attentional modulation, the correlation analysis revealed no significant relationship (see below). One possible reason for the lack of significance is that the duration discrimination task likely involves additional cognitive processes, such as working memory (for example, holding three sounds in mind and identifying the longest) and processing speed, which make it more complex, whereas offset encoding is primarily driven by sensory neural mechanisms. Furthermore, although sound-offset encoding is an important determinant of duration discrimination, other factors, such as sound-onset

encoding and the encoding of sustained sound activity, may also affect behavioural DDT performance. We added this active/passive offset ratio in our correlation analysis (see revised Table 2), since it is a valid point and readers might be curious about it.

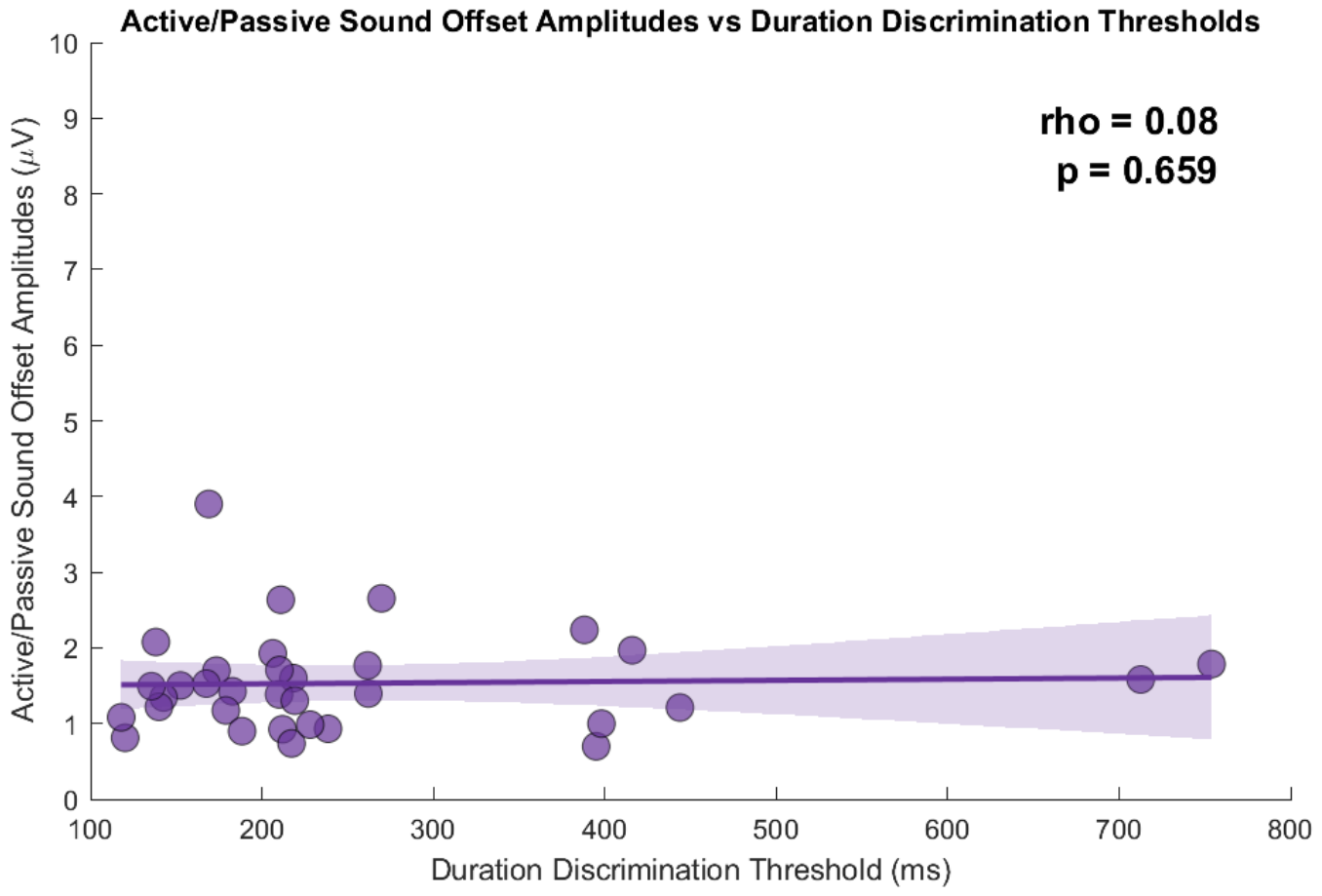

5. Line 163: update placeholder by correct file number.

RESPONSE: As you suggested, we have updated the placeholder.

Referee #2:

Review: "Sound offset encoding is related to speech-in-noise perception at sentence level in older adults" by Hasan Colak et al.

This study describes EEG measurements of auditory-evoked ON- and OFF-responses in a group of 34 older adults (6-81 years of age) with near-normal hearing. The responses were elicited by 1-2-s, 75-dB SPL noise bursts, separated by 1-2 s silent gaps, and were recorded in both active and passive listening conditions. The aim was to test whether OFF-responses could be measured at a single-subject level and whether their size, measured between the FCz and combined P9/10 channels, is significantly associated with gap detection, duration discrimination, or speech-in-noise (SiN) reception performance. The results showed (i) significant OFF-responses in every subject; (ii) attentional effects (difference between active and passive recording conditions) in the OFF- but not ON-response; (iii), significant positive association between active OFF-response size and sentence-, but not word-in-noise performance. Based on these results, the authors suggest that the OFF-response may be a "potential clinical measure of auditory cognition relevant to speech-in-noise listening".

This study has several major shortcomings, which mean that the overall knowledge advance created is likely minimal and, in fact, the study results may be inadvertently misleading. A major problem is that it is not clear how exactly the study was motivated; what questions was it intended to answer, or what hypotheses was it designed to test?

RESPONSE: Dear Reviewer,

Thank you for taking the time to read our paper and for your helpful suggestions to improve the study. We believe our work had a clear motivation, and the current study was designed specifically to address these aims.

First, we aimed to determine whether the sound offset response can be reliably recorded in all participants. We agree that this is not a novel finding. However, replication is essential to demonstrate that sound offset responses could one day be used in clinical settings. It's important to show that the response can be measured consistently so we know what's normal versus abnormal, and whether it could help identify people with certain hearing difficulties. We also needed to confirm that individuals consistently show offset responses before moving on to the next aims.

Second, we investigated whether sound offset responses can be modulated by attention using a passive listening condition and an active listening task in which participants focused on the beginning and end of the sound. This is a less studied aspect of offset responses, and our findings likely indicate that a top-down mechanism is involved in sound offset encoding.

Third, we tested whether sound offset responses relate to everyday speech-in-noise performance. Some previous work (e.g., Kopp-Scheinpflug et al., 2018) has suggested there could be a link, but to our knowledge no one has directly tested this yet. We also know from previous research that temporal processing contributes to speech-in-noise perception. We therefore considered it a reasonable question to ask whether the sound offset component of temporal processing might specifically contribute to this ability. To address this, we included two speech-in-noise tasks, one at the word level and one at the sentence level, allowing us to explore this question.

Considering your feedback, we have revised our paragraph as follows to clarify the aims of this study for the reader in the paper:

"The aims of this study are to determine whether brain responses to sound offsets can be reliably recorded at the single-subject level in older adults and to compare them with onset responses. Our goal is to understand whether offset responses are robust in this population and to pave the way for potential clinical applications and further investigation in auditory cognitive disorders. We also aim to assess whether onset and offset responses are modulated by task demands by comparing passive listening with an active duration discrimination task, allowing us to test the role of attention. Finally, we investigate the relevance of onset and offset responses to everyday listening by examining their relationship with word- and sentence-in-noise performance in older adults."

The auditory-evoked OFF-response has been subject to extensive research, much of which took place in the previous century. This has shown that the occurrence of an OFF-response depends crucially on the eliciting sound duration (e.g., Hillyard & Picton, 1978, "ON and OFF components in the AEP"; Song et al., 2024, "A new function of offset response in the primate auditory cortex: marker of temporal integration"), and that, when the eliciting sound is more than a few 100 ms long, an OFF-response generally occurs just as reliably as the ON-response. Therefore, the first finding of "robust [...] offset responses at a single-subject level" is not in any way novel or surprising.

RESPONSE: Thank you for your comment. We agree that robust sound-offset responses are not a surprising finding. However, it was important to demonstrate this in our sample, as this study is one of the few that examine sound offset responses in older adults. Regarding the reliability of offset responses, much of the earlier work was conducted for experimental purposes, often using small changes to stimulus characteristics and very limited sample sizes. For example, in the study by Hillyard & Picton (1978), experiment 1 included only 10 young adults and experiment 2 included just 5 adults. Findings from such small samples do not necessarily generalize to our older adult participants. Furthermore, many previous studies of sound offset responses either involved similarly

small human samples or focused primarily on animal models. We therefore felt it was important to confirm the reliable recording of offset responses in our specific sample. This reliability is a necessary first step before examining attentional modulation, relationships with speech-in-noise performance, and the potential use of offset responses as a clinical biomarker.

We acknowledge that sound offset amplitudes can depend on stimulus duration, and we believe this may help explain why we did not observe a relationship between word-in-noise (WIN) test scores and sound offset amplitudes in this study. We have included this in the discussion as follows:

"A study by Hillyard and Picton (1978), as well as an animal study by Solyga and Barkat (2021), showed that cortical sound offset amplitudes increase as the duration of the sound increases. This raises the possibility that randomly varying sound duration from 1 to 2 seconds in our task may not be representative of the brief durations of words in the WIN test."

The second finding, that the OFF-response was larger in the active than passive listening condition, whereas the ON-response was statistically unchanged, conflicts with an extensive body of previous research showing that the ON-response can be subject to substantial attentional modulation (typically, the ON-response approximately doubles in size when attention is actively focussed on the eliciting sound). This finding is thus specific to the current experimental design carries no general validity or value. Looking at the current data, it is interesting to note that the ON-response appeared to be slightly, but largely consistently, smaller in the active compared to passive listening condition. A similar inverted relationship of the ON-response size was also observed by Hillyard & Picton (1978) in a study that varied the OFF-response size by varying sound duration, perhaps indicating some form of asymmetric functional interdependence between the two types of responses (whereby a larger OFF-response causes a smaller ON-response but not vice versa).

RESPONSE: Thank you for your comment.

We would like to clarify a couple of issues first. In our original analysis, the comparison of onset and offset responses between active and passive listening conditions was based on an unpaired data structure. In this rebuttal, we reanalysed the data using a paired design, following the suggestions of reviewers 1 and 3. This paired analysis revealed that onset responses in the active condition were smaller in amplitude than in the passive condition. This was indeed an unexpected finding. We believe it can be explained by the fixed task order, where all participants completed the passive condition first, followed by the active condition (in this revision, we also corrected an error in our original description of the task order. This can be verified by checking the EEG recording timestamps in the uploaded datasets, see above). To avoid any confusion, we would like to clarify that all participants completed the passive condition first, followed by the active condition. The reduced onset amplitudes in the active condition may reflect adaptation or habituation due to repetitive sound presentation. To successfully complete a duration discrimination task, both onset and offset encoding are required. During the sound, attentional load is likely to increase as it approaches the end. If the active task does enhance onset responses, this effect may have been counteracted by adaptation or habituation. In contrast, the larger offset responses observed in the active condition compared to passive listening could be due to the increasing attentional demands toward the end of the sound. We have revised the relevant paragraph in the discussion section accordingly, as given below.

"The present work provides evidence for task-dependent modulation in temporal processing, demonstrating that offset responses were significantly larger in the active listening condition compared to the passive condition, whereas onset responses were significantly smaller in the active listening condition than in the passive condition. This finding is partly consistent with results of previous work by Ali et al. (2025), which reported increased offset responses but no task-related change in onset responses. The smaller onset responses observed in the active task in our study were

indeed unexpected. One plausible explanation relates to our design, in which participants always completed the passive EEG condition first, followed by the active task. Reduced onset responses in the active condition may therefore reflect stimulus adaptation triggered by repeated sound presentation. The increase in offset responses during active listening suggests a potential attention mechanism involved in sound-offset encoding, where a top-down (e.g., endogenous) process also becomes important. More specifically, following sound onset, attentional load, sustained attention in particular, may increase over the course of the stimulus in preparation for detecting the sound offset, as accurate offset detection is critical for estimating sound duration. This increased attentional demand toward the sound offset may contribute to the amplified offset responses in the active listening condition."

What we take from this is that sound-offset responses are task-dependent and can be modulated with a suitable task. For this reason, we find it difficult to agree with your statement that 'this finding is thus specific to the current experimental design and carries no general validity or value.' All findings are, to some extent, dependent on their experimental design. By this reasoning, one could also argue that studies showing doubled responses with attention, as you referenced, carry no general validity, since we do not replicate such a finding (see also Ali et al., 2025, https://doi.org/10.1101/2025.07.23.666338). In our case, we deliberately chose a duration discrimination task as an ideal paradigm to elicit strong onset and offset responses. We agree that further research is needed to examine how offset responses are affected by different tasks, but we believe our results provide sufficient evidence to conclude that offset responses are task-dependent.

Your final point about a possible asymmetric functional interdependence between onset and offset responses is indeed interesting. While any argument on this would currently be speculative, we agree it is still worth mentioning. We have therefore added an additional paragraph focusing on this idea (see below), outlining potential mechanisms that could explain such an effect.

"An alternative, more speculative explanation for the smaller onset amplitudes in the active condition could involve an asymmetric functional interdependence between onset and offset responses. Hillyard and Picton (1978) reported a similar inverted relationship, where manipulations that increased offset responses were accompanied by reduced onset responses. At the neural level, although onset- and offset-sensitive populations are partly distinct, some auditory cortical neurons can respond to both onset and offset cues, albeit through different sets of synapses (Scholl, Gao, & Wehr, 2010; Volkov & Galazjuk, 1991). When offset encoding demands are higher, these dual-responsive populations may preferentially contribute to the offset response, which could in turn diminish their contribution to the onset response at the scalp level. One possible mechanism is neural refractory effects, where recent offset-related activity transiently limits the ability of these neurons to generate a strong onset response. Another possibility is a redistribution of processing resources, in which attentional mechanisms bias neural activity toward the task-relevant offset cue at the expense of onset encoding. In this sense, greater offset responses may occur at the expense of smaller onset responses, reflecting a trade-off rather than an absolute absence of attentional modulation of onset encoding. Further research is required to test whether offset responses can indeed increase at the cost of onset responses and, if so, to identify the mechanisms underlying such a one-way trade-off."

The remaining findings are purely correlational, which, without a clearly stated prior aim or question, and given the relatively small sample size, is highly problematic, particularly, because the authors only discuss findings that survive their relatively stringent family-wise error threshold (Bonferroni correction for 45 comparisons; where exactly does this number come from?). I presume that the authors' expectation of a correlation with SiN performance was predicated on the idea that the OFF-response may play a functional role in the identification of stop consonants, where the initial burst and subsequent release or voicing onset are separated by a short silent gap, the duration of which

determines the perceived consonant category (as hypothesized, e.g., by Kopp-Scheinpflug et al., 2018). Whilst Dr Kopp-Scheinpflug's argument may be valid at the microscopic (single-neuron) level, it almost certainly isn't valid at the level of the gross, long-latency OFF-responses measured with EEG and MEG. This is because, as I pointed out above, the occurrence of these OFF-responses depends on the preceding sound duration, with an OFF-response occurring only when the preceding sound is several 100's of ms long. Therefore, OFF-responses can't be functionally essential for stop-consonant identification, which human listeners can happily accomplish even when consonants are presented in isolation, and thus the silent gap is preceded by only a very brief burst. For the reader, it thus remains unclear as to why a correlation between the OFF-response size and SiN performance should be expected and, assuming the observed correlation is true, how such correlation may be brought about. On the other hand, the more obvious correlations of OFF-response size with gap detection and duration discrimination performance, which the authors almost certainly expected to find at the outset of their study, are barely mentioned at all, merely because they don't survive the family-wise error criterion. This epitomizes the power problem in small correlational studies with multiple comparisons (Yarkoni, 2009, "Big correlations in little studies"; see also Ioannidis, 2005, "Why most published research findings are false"; and Button et al., 2013, "Power failure: why small sample size undermines the reliability of neuroscience"): To guard against family-wise type-I error, such studies set perniciously high type-I error thresholds (of $p < 0.05/M$, where M is the number of comparisons conducted). As a result, the probability of detecting a 'true' effect (i.e., an effect that is actually present) in each single comparison becomes very small, even if the actual/true effect size is comparatively large, thus leading to an inflated type-II error rate (i.e., likelihood of missing a true effect), whilst at the same time inflating the observed effect size in those comparisons that manage, by chance, to surpass the significance threshold.

RESPONSE: Thank you for pointing out this concern. As stated above, the final aim of this study was to examine whether sound-offset encoding is relevant to everyday speech-in-noise perception. We were particularly interested in correlations between sound-offset amplitudes and speech-in-noise test scores. We agree that the remaining correlations in the table may appear crowded, as they include relationships we were not specifically investigating (e.g., PTA vs. Duration Discrimination threshold). Our initial reason for presenting a full correlation matrix, where every variable was correlated with every other variable, was to allow readers to see the broader relationships between behavioural measures, EEG responses, and speech-in-noise performance at the same time. As advised by Reviewer 3, we have updated the correlations and now only show the relationships between electrophysiological findings and behavioural tests.

We originally had 10 variables in the table, giving $C(10, 2) = 45$ comparisons. In this rebuttal, we have revised the correlation analysis by applying the Benjamini–Hochberg correction for multiple comparisons and by reducing the number of variables in the table, as suggested by Reviewer 3.

We expected to see a relationship between speech-in-noise perception and sound-offset encoding based on the hypothesised role of the offset response in stop-consonant identification (Kopp-Scheinpflug et al., 2018). We understand your concern that this argument may not hold at the gross level of EEG or MEG, where an OFF-response typically depends on the preceding sound duration and may require around 100 ms to emerge clearly. The reason for this is that shorter durations can lead to temporal overlap between onset and offset responses, making it difficult to separate them. However, just because we cannot fully disentangle onset and offset contributions at the gross level does not mean that offset encoding has no role in speech-in-noise perception, nor does it imply that offset mechanisms at the gross and neuronal levels are entirely different. Therefore, we do not believe this argument is strong enough to invalidate the proposed role of offset responses in speech-in-noise processing.

There may also be additional roles for sound-offset responses in speech-in-noise perception. For example, offset responses may contribute to speech segmentation or to detecting "dips" in fluctuating background noise, as discussed in the manuscript. In real-world listening, background noise is often discontinuous (similar to our tasks), with brief silent intervals. A well-functioning offset-encoding mechanism could help listeners exploit these silences, improving speech processing under more favourable SNR conditions.

We agree that our effect sizes may be overestimated due to the small sample. While the true effect of the relationship between offset responses and sentence-in-noise performance may be smaller than observed here, we believe the effect size is still large enough to suggest a meaningful link at the sentence level. Further research with larger samples will be necessary to replicate these findings and clarify the relationship between offset encoding and speech-in-noise perception at both the word and sentence levels, thereby reducing the risk of effect inflation. Based on your comment, we have expanded the limitations section to emphasise that our findings should be interpreted with caution.

"Second, the correlational analyses were exploratory and conducted in a relatively small sample, making the results susceptible to both inflated effect sizes and reduced power to detect true effects, as is the case in all studies using small sample sizes (Button et al., 2013; Yarkoni, 2009). Although we controlled for multiple comparisons using the Benjamini–Hochberg procedure, the possibility of false positives remains, and these findings should be interpreted with caution. Larger studies are needed to better establish the role of sound-offset encoding in speech-in-noise perception and to clarify the underlying mechanisms."

As a result of the latter effect of low power (i.e., overestimation of the effect size of significant effects), the idea that the OFF-response may have potential as a clinical marker for speech-relevant cognitive functions is widely over-optimistic. The current data suggest that the OFF-response size in a single EEG electrode (PCz vs P9/10) explains as much as one third (33%) of inter-individual variance in sentence-in-noise perception in older, albeit near-normal hearing listeners. Given the vast degree of variance in both SiN performance and long-latency cortical auditory-evoked potentials, particularly when measured within individual electrodes (i.e., not taking into account variation in response scalp distribution), I would expect the real effect (if there is one) to be more of the order 1% or even less.

RESPONSE: Thank you for your comment. We agree that the effect size may be overestimated due to the small sample, and the true effect could indeed be smaller than what we observed. However, we do not agree that the potential clinical relevance of the OFF-response to speech-in-noise perception is 'widely over-optimistic.' While our study may overestimate the role of sound offset encoding in speech-in-noise perception, this question can only be resolved through well-designed future studies, rather than opinions not grounded in empirical evidence.

We suspect that there may be a typo where you refer to the electrode as PCz (we assume you meant FCz). We focused on this electrode because offset responses are typically largest at FCz (Ali et al., 2025, https://doi.org/10.1101/2025.07.23.666338). Furthermore, we do not see how the figure of 33 percent inter-individual variance was derived. Our analysis supports the possibility of a clinically relevant association between sound-offset encoding and speech-in-noise performance. From a clinical standpoint, a feasible measure should ideally be obtainable from a single electrode, preferably one closest to the neural source of interest (in this case, FCz), rather than requiring whole-scalp recordings, which are more time consuming and costly.

Given the relatively low-level (automatic) nature of the N1-P2, the main components of the OFF-response, I would also assume that any true association between OFF-response size and SiN performance is artefactual, or trivial, in nature, likely caused by variation in low-frequency hearing thresholds (subclinical in the current study). Calculating the partial correlation between OFF-response

size and SiN performance with respect to average hearing thresholds (the so-called PTA, which the current authors calculate across the entire audiometric range from 0.25 to 8 kHz) does not necessarily mean that a potential confounding effect of hearing thresholds has been successfully precluded. This is because, firstly, the effect of hearing thresholds on OFF-response size, SiN performance or both may be non-linear and thus inadequately accounted for by linear partialling out. More importantly, averaging hearing thresholds to a single, wideband PTA measure may have diluted the effects of low-frequency thresholds, which, even though the stimulus was a wideband noise, would be expected to have the greatest effects on long-latency cortical auditory-evoked responses, such as the OFF-response (this is due to the layout of the cortical tonotopic map, which favours low-frequency response contributions).

RESPONSE: Thank you for pointing this out. We understand the concern that low-frequency hearing thresholds may be driving the observed association. To address this, we conducted additional partial correlation analyses between sentence-in-noise scores and offset amplitudes, controlling separately for low-frequency PTA (0.25, 0.5, 1 kHz), mid-frequency PTA (1, 2, 4 kHz), and high-frequency PTA (6 and 8 kHz). The results are shown below, and none indicated that the association was driven by hearing thresholds.

We also recognize the concern that linear partialling out may not be ideal if a non-linear relationship exists. However, it seems less likely that the relationship between sentence-in-noise and offset amplitudes is driven by low-frequency PTA, as there was only limited variation among participants at low frequencies (mean:7.99, SD: 6.25). To further address this issue, we created a subsample including participants with low-frequency PTA values between 0 dB (the minimum in our dataset) and 10 dB. This group comprised 25 out of 34 participants, showing the small variability in low-frequency hearing thresholds in our sample. We re-ran the same correlation analysis in this well-controlled subsample and still observed a significant relationship between sentence-in-noise scores and offset amplitudes, with a large effect size (see below). Overall, these analyses suggest that the observed association is unlikely to be driven by hearing thresholds in the current sample.

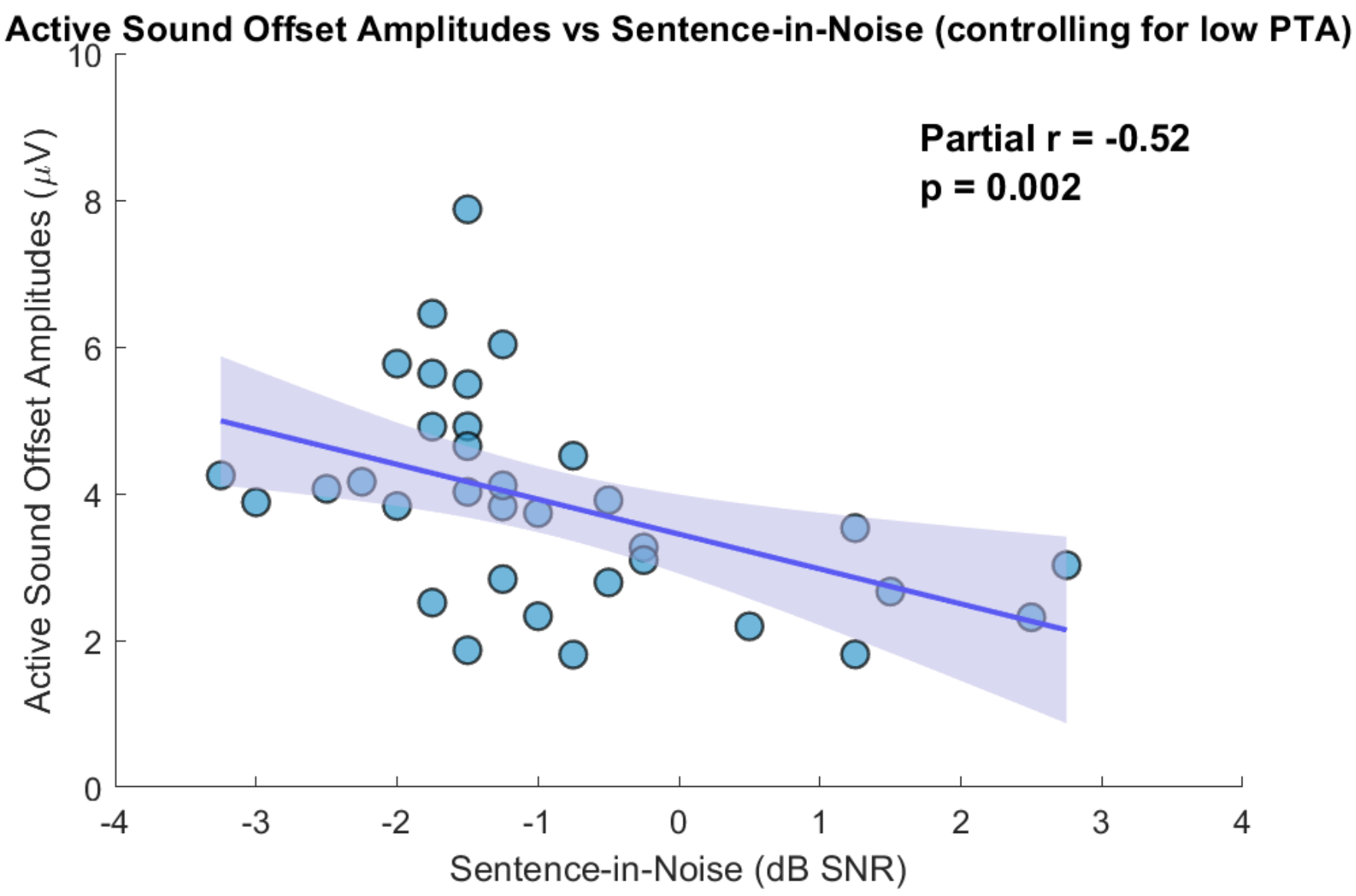

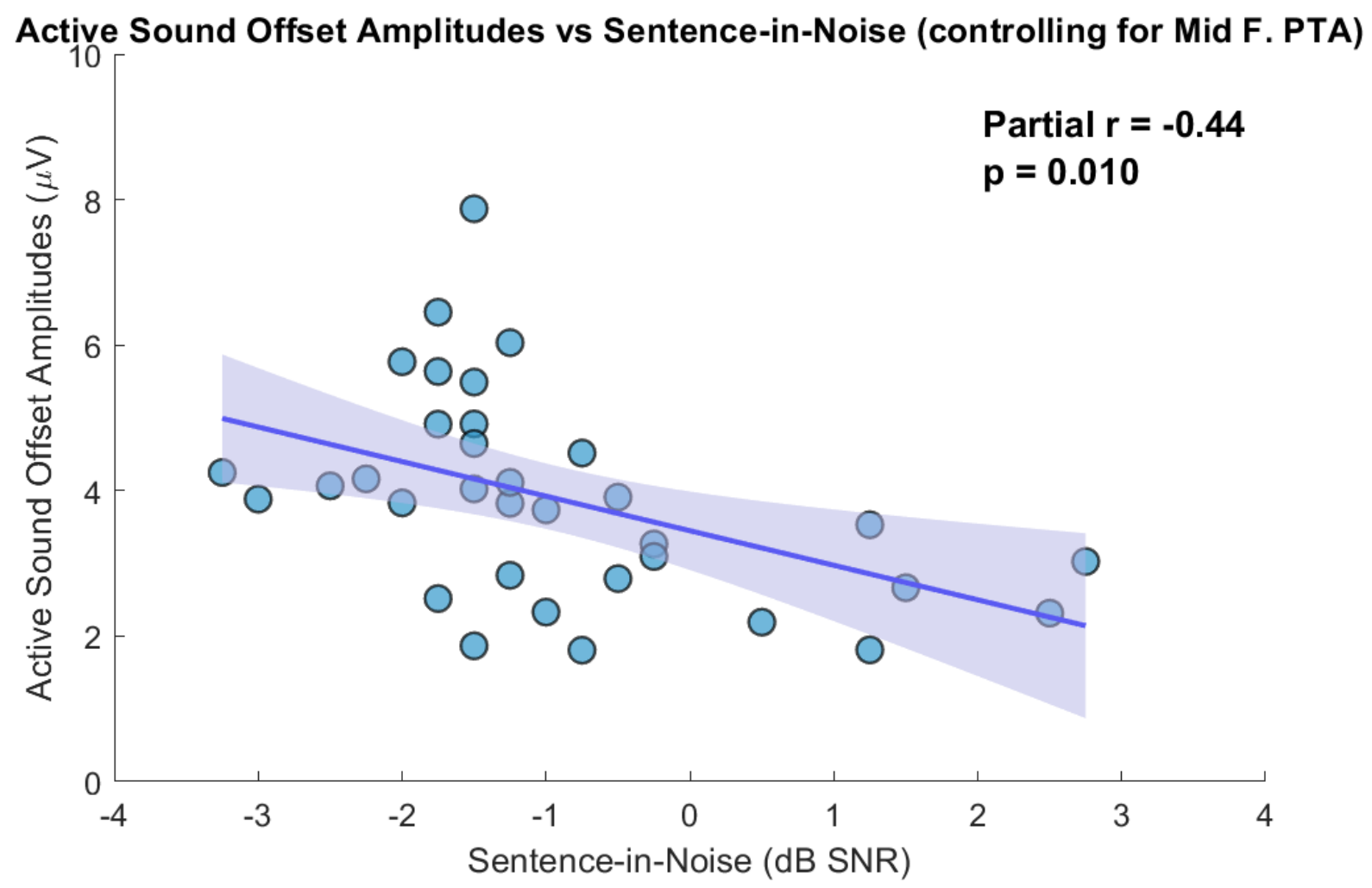

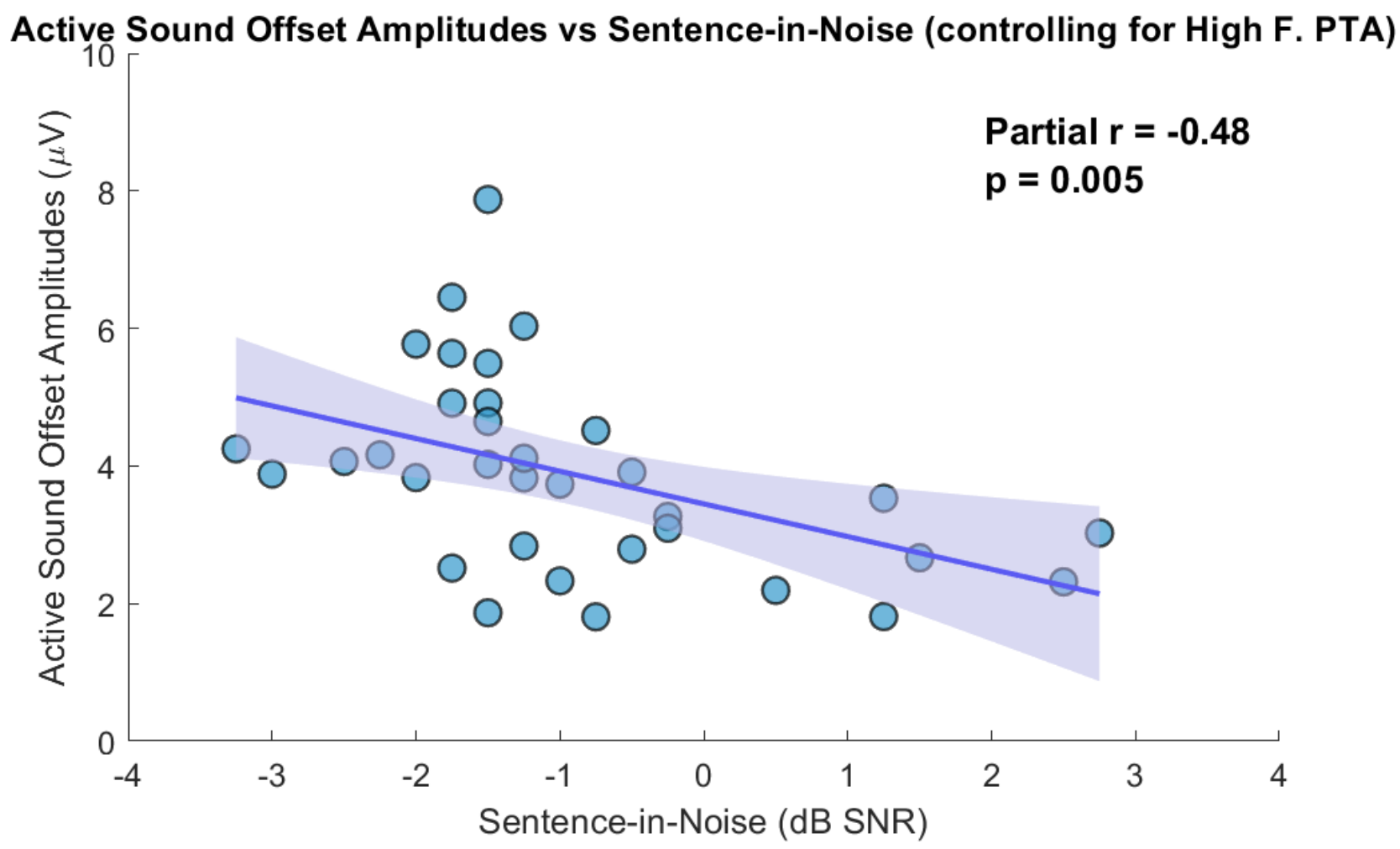

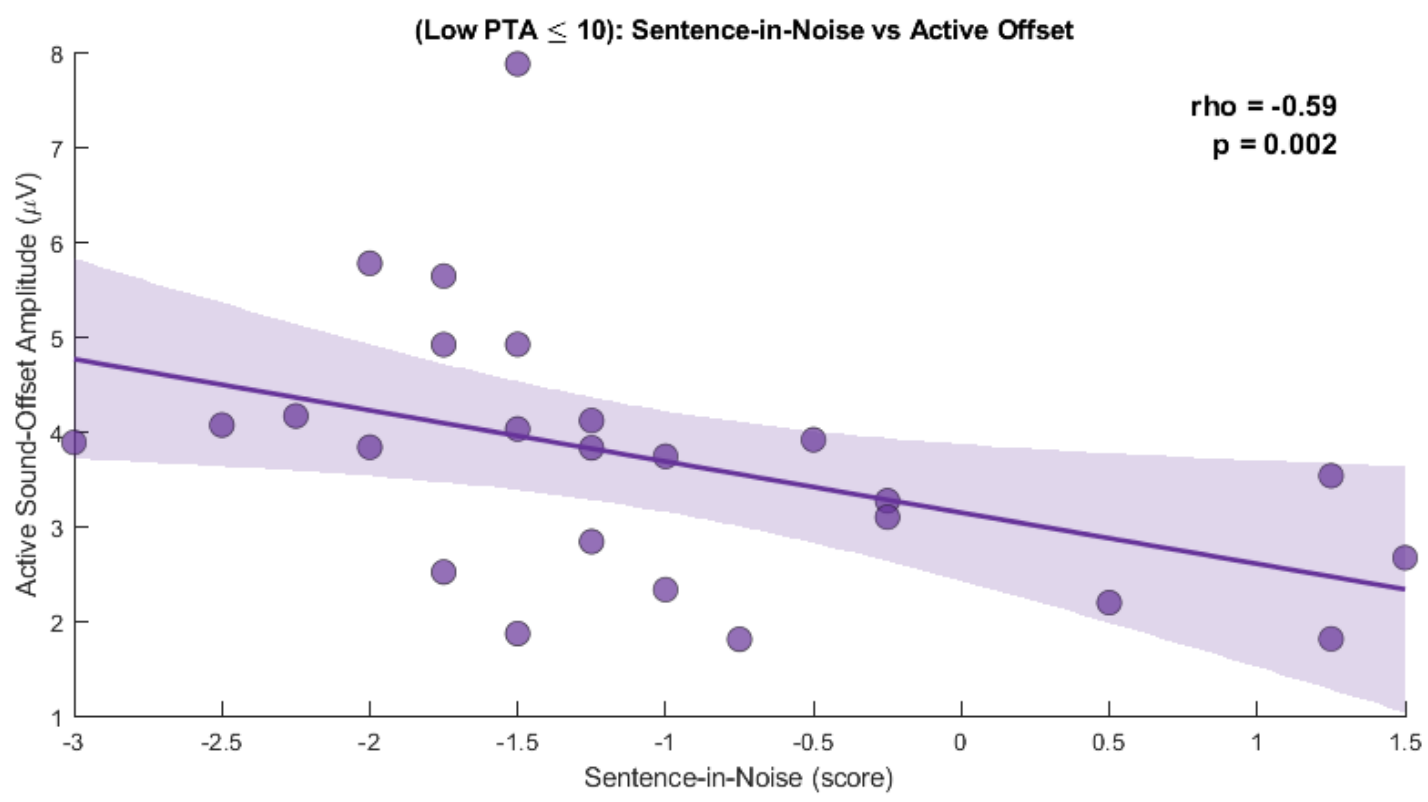

Overall, I feel that the authors should focus much more on what they originally set out to achieve, rather than moulding the story to suit the 'significant' results.

RESPONSE: Thank you for your comments, time, and suggestions to improve our work. The current study design is quite specific and does not allow for moulding the results. We specifically designed a

task to examine how an onset- and offset-relevant task affects offset responses. In addition, we included two speech-in-noise tasks, at both the word and sentence levels, to directly assess whether offset encoding is related to speech-in-noise perception. Therefore, the design itself does not allow us to mould or manipulate the significant findings.

Referee #3:

The work presented here measured onset and offset responses in older listeners, and related those to a number of behavioral performance measures: duration discrimination, gap detection, word in noise and sentence in noise. The most important result (in my eyes) is that offset responses can be robustly measured, are task-sensitive (larger in active than in passive listening), and show some correlation with behavioral performance, in particular sentence in noise performance.

However, there are significant weaknesses in the data analysis and the way conclusions are derived from the data. The most important is that repeated assertion that there was no task dependence in the onset responses. This claim is directly falsified by the data in Fig. 4B. I would guess that the tests showing lack of significant difference were unpaired. However, the data is paired (active and passive P2-N1 measured in the same subject), and across-subject variability is considerable (as obvious from Fig both panels of Fig. 4), artificially reducing the significance of unpaired tests. A paired comparison between the active and passive onset P2-N1 values give a strongly significant difference (even the weak sign test, which I could perform with the data in Fig. 4B, gives a highly significant result). Since this is a crucial part of the claims in this paper, this manuscript as it stands is wrong.

RESPONSE: Dear Reviewer, Thank you for taking the time to read our paper and for your helpful suggestions to improve the study.

We would like to clarify that in the original submission, the statistical comparison between active and passive onset responses was indeed based on an unpaired analysis. Following the comments from you and other reviewers, we have reanalysed the data using a paired approach, given that active and passive responses were measured within the same participants. This paired-sample analysis revealed that onset amplitudes in the active condition were significantly smaller than in the passive condition.

This was indeed an unexpected finding. We believe it can be explained by the fixed task order, where all participants completed the passive condition first, followed by the active condition (in this revision, we also corrected an error in our original description of the task order. This can be verified by checking the EEG recording timestamps in the uploaded datasets, see above). This order could have introduced adaptation or habituation effects that affected the onset responses in the active block. Furthermore, the duration discrimination task we used likely places a greater attentional load toward the end of the sound, as the end of the sound determines which stimulus is longer, rather than at the onset. This focus on the sound's ending could explain why the offset response increased in the active condition, while we did not observe the same effect in the onset responses.

We have revised the manuscript to present the paired-sample results in place of the original unpaired analysis. We have also updated the wording thorough the paper accordingly to reflect the paired analysis results and possible mechanisms underlying the observed results (see below).

"The present work provides evidence for task-dependent modulation in temporal processing, demonstrating that offset responses were significantly larger in the active listening condition compared to the passive condition, whereas onset responses were significantly smaller in the active

listening condition than in the passive condition. This finding is partly consistent with results of previous work by Ali et al. (2025), which reported increased offset responses but no task-related change in onset responses. The smaller onset responses observed in the active task in our study were indeed unexpected. One plausible explanation relates to our design, in which participants always completed the passive EEG condition first, followed by the active task. Reduced onset responses in the active condition may therefore reflect stimulus adaptation triggered by repeated sound presentation. The increase in offset responses during active listening suggests a potential attention mechanism involved in sound-offset encoding, where a top-down (e.g., endogenous) process also becomes important. More specifically, following sound onset, attentional load, sustained attention in particular, may increase over the course of the stimulus in preparation for detecting the sound offset, as accurate offset detection is critical for estimating sound duration. This increased attentional demand toward the sound offset may contribute to the amplified offset responses in the active listening condition."

While I commend the authors for supplying what seems to be their full dataset, this is insufficient for checking the correctness of the analysis - they need to supply also the analysis routines they used, and even better, the scripts used to generate the figures and run the statistics. Furthermore, their dataset lacks any documentation, making it effectively useless.

RESPONSE: As you advised, we have shared our analysis scripts, including those used to generate the figures. These are available via the same OSF link. We have also prepared a readme file to assist users.

Major comments:

The introduction doesn't make sense in the context of the paper. The main result is the different effects of active vs. passive listening on the offset responses, but the effects of attention on offset responses is not discussed at all, or the robustness and potential source populations for offset-dependent ERP components is not discussed. Instead, the intro speaks mostly about the importance of offset responses to speech understanding, which is not the only or even main point of the paper.

RESPONSE: Thank you for pointing this out. Although the speech-in-noise relevance of the offset response is an important point in the paper, we agree that similar emphasis should be placed on the task dependence of the offset responses. As you suggested, we have expanded the introduction section accordingly.

"The earlier attempts to record EEG onset and offset responses in the human brain have revealed asymmetries between these responses, such as smaller and slower offset responses compared to onset responses (Hillyard & Picton, 1978; Pantev et al., 1996). Hillyard and Picton (1978) also reported that sound-offset responses depend on stimulus duration, with offset amplitudes increasing as the duration becomes longer. Offset EEG responses show morphological differences compared to onset responses. For example, the P1 component is consistently absent in offset responses, whereas onset responses typically follow the P1–N1–P2 waveform (Pantev et al., 1996; Pratt et al., 2008). At the cortical level, offset responses are generated predominantly in the bilateral auditory cortex on the supratemporal plane, particularly in Heschl's gyrus and the planum temporale, similar to onset responses (Hari et al., 1987; Pantev et al., 1996). Pratt, Bleich and Mittelman (2005) studied the effect of attention on the sound-offset response and found no evidence of attentional modulation in the earlier N1 component. Rather, Harris et al. (2012) reported an attentional modulation in the later N2 component of the sound offset response. However, many previous studies overlooked the role of stimulus duration, which may have led to interference between onset and offset responses, making it difficult to clearly separate the two."

The references in the introduction are very partial - important animal work is missing (e.g. the work of the group of Mike Wehr).

RESPONSE: Thank you for bringing this to our attention. We have now included Dr. Wehr's work in the introduction, along with other relevant studies in the field (see below).

"At the cortex level, both onset and offset responses are driven by transient synaptic inputs, but they typically arise from largely nonoverlapping sets of synapses with different frequency tuning, indicating that onset and offset responses are partly distinct yet coexisting mechanisms within the auditory cortex (Scholl, Gao, & Wehr, 2010)."

Analysis of Table 2: The Bonferroni correction here is based on 45 comparisons. However, only a much smaller number of correlation coefficients here are really interesting. The matrix correlates everything with everything, but the interesting correlations are only those that pertain to the behavioral measures vs. electrical measures - these are the crucial tests of this paper. There are 5 of each, giving in total only 25 interesting comparisons. More importantly, these values are highly correlated among themselves, and certainly don't fit the assumption of the Bonferroni correction (independence of the tests that are being conducted). Thus, this approach doesn't make sense. There are other correction schemes (e.g. Benjamini-Hochberg) and other approaches to the study of correlation structure (e.g. factor analysis and related methodologies) that make much more sense here.

RESPONSE: We agree that the initial correlation table was somewhat crowded and included correlations that were not of primary interest. We also agree that the Benjamini–Hochberg (B-H) procedure is more appropriate for the current study. As you suggested, we applied the B-H procedure to correct our correlation analysis and updated the table. In the revised version, we used the 25 correlations you recommended and also included 5 additional correlations looking at the relationship between the active/passive offset ratio and behavioural measures, as suggested by Reviewer 1 (see table 2 below).

"**Table 2.** Spearman correlation coefficients (rho), p values, and Benjamini–Hochberg FDR-corrected q values for associations between behavioural measures (rows) and EEG onset/offset responses (columns).

	**Active Offset**	**Active Onset**	**Passive Offset**	**Passive Onset**	**On/Off Active**	**Active/Passive Offset ratio**
**GDT**	rho = 0.03	rho = -0.09	rho = -0.19	rho = -0.25	rho = -0.03	rho = 0.17
	p = 0.862	p = 0.632	p = 0.288	p = 0.160	p = 0.854	p = 0.330
	q = 0.862	q = 0.732	q = 0.598	q = 0.494	q = 0.862	q = 0.611
**DDT**	rho = -0.13	rho = -0.25	rho = -0.19	rho = -0.24	rho = -0.09	rho = 0.08
	p = 0.460	p = 0.148	p = 0.269	p = 0.171	p = 0.615	p = 0.659
	q = 0.732	q = 0.494	q = 0.598	q = 0.494	q = 0.732	q = 0.732
**WIN**	rho = 0.17	rho = -0.08	rho = 0.41	rho = -0.18	rho = -0.22	rho = -0.09
	p = 0.347	p = 0.659	p = 0.016	p = 0.299	p = 0.221	p = 0.623
	q = 0.611	q = 0.732	q = 0.096	q = 0.598	q = 0.553	q = 0.732

**SIN**	**rho = -0.58**	rho = -0.41	rho = -0.42	rho = -0.25	rho = 0.06	rho = -0.23
	**p < 0.001**	p = 0.016	p = 0.015	p = 0.156	p = 0.720	p = 0.181
	**q = 0.010**	q = 0.096	q = 0.096	q = 0.494	q = 0.771	q = 0.494
**PTA**	rho = -0.09	rho = -0.32	rho = -0.45	rho = -0.09	rho = -0.11	rho = 0.12
	p = 0.597	p = 0.064	p = 0.008	p = 0.627	p = 0.528	p = 0.514
	q = 0.732	q = 0.321	q = 0.096	q = 0.732	q = 0.732	q = 0.732

I'm a bit surprised that the gap detection performance showed such poor correlation with the speech in noise tests. Some clinical literature suggests that gap detection should correlated with speech performance.

RESPONSE: We agree that a correlation between gap detection and speech-in-noise measures could be expected. However, the literature shows inconsistencies in this relationship across studies. Our group has prepared a brief review on the relationship between temporal processing measures and speech-in-noise performance. Below, we have included a short paragraph illustrating these inconsistencies in the relationship between gap detection and speech-in-noise tests. It is possible that, unlike the within-channel gap detection test used in our study, between-channel gap detection tests may correlate more strongly with speech-in-noise perception.

“Studies comparing the two types of GDT are limited. In clinics, within-channel GDT tests have been more commonly used, such as the Gap-in-Noise test (GIN, Musiek et al., 2005), the Random Gap Detection Test (RGDT, Keith, 2000), the Adaptive Test of Temporal Resolution (this test has a component of between-channel detection, Lister et al., 2006), Auditory Fusion Test-Revised (McCroskey & Keith, 1997). They are used as a way of assessing temporal resolution that can inform the diagnosis of auditory processing disorder. However, in terms of the predictability of SIN listening, the literature suggests that within-channel gap detection does not consistently reflect the performance of commonly used SIN performance. An early psychoacoustic experiment looking at temporal acuity, sentence-in-noise, and reverberation found a strong correlation between the two measures (Irwin & McAuley, 1987). However, the study was conducted with a very small sample (8 participants) so the results might not be reliable. Similarly, a behavioural correlation was established by Feng et al. (2010) with native Mandarin speakers with high-frequency hearing loss. However, the researchers cautioned that the data showed large individual variations and needed to be validated. A significant correlation was found between RGDT and sentence-in-babble measures in CI patients (Blankenship et al., 2016). Heeke et al., (2018), on the other hand, found a negative correlation between RGDT and HINT threshold measures and the correlation was not significant after correction for multiple comparisons. While older people showed lower gap-detection ability, their SIN ability still held up, and no significant correlation was found between GIN and R-SPIN (De Metropoliset al., 2021).This result was consistent with the study conducted by Hoover et al., (2015), who found non-significant correlations between GIN and SIN perception in normal hearing people, and Cesur & Derinsu (2020) in CI users. Snell & Frisina (2000) proposed that the relationship between the SIN measures and gap detection might be modulated by age. They investigated younger and older age groups and found a significant correlation between SIN perception and gap thresholds in younger participants but no association in the older age group. However, the opposite results were found by the same group when they attempted to replicate the result (Snellet al., 2002). Overall, there is little consistency on the literature reporting an association between SIN and gap detection measures.”

Minor comments:

Discussion: around lines 380, critique of the ecological validity of pure tones: I don't think that 1-s lone white noise is more ecological than pure tones. This is not a serious argument and I would remove it.

RESPONSE: We agree that a white-noise stimulus is not as ecological as many natural surrounding sounds. But, the key point here is to emphasize its broadband structure, which makes it more similar to speech sounds.

Lines ~400, offset responses and change detection: Awwad et al. (2021) showed that offset responses, as well as some sort of omission responses, occur in awake but not anesthetized rats. Maybe worth mentioning here, this has the same flavor.

RESPONSE: Thank you for bringing this to our attention. We have added this study to the relevant paragraph in the discussion.

"In line with this idea, Awwad et al. (2023) reported omission responses resembling offset responses in unanaesthetised, but not in anesthetized rats."

Line 163: the ethics committee name is corrupted.

RESPONSE: We have now replaced it with the full name of the ethics committee.

Line 289 refers to supplementary material 1, I couldn't locate it.

RESPONSE: It has been uploaded again with this revision.

Dear Dr Colak,

Re: JP-RP-2025-289104R1 "**Sound Offset Encoding is Related to Speech-in-Noise Perception at Sentence Level in Older Adults**" by Hasan Colak, Xiaoxuan Guo, Adèle Simon, Ester Benzaquén, William Sedley, Jennifer F. Linden, and Timothy D. Griffiths

Thank you for submitting your manuscript to The Journal of Physiology. It has been assessed by a Reviewing Editor and by 3 expert referees and we are pleased to tell you that it is acceptable for publication following satisfactory revision.

The referee reports are copied at the end of this email.

Please address all the points raised and incorporate all requested revisions or explain in your Response to Referees why a change has not been made. We hope you will find the comments helpful and that you will be able to return your revised manuscript within 4 weeks. If you require longer than this, please contact journal staff: jp@physoc.org.

Your revised manuscript should be submitted online using the link in your Author Tasks Link Not Available. This link is accessible via your account as Corresponding Author; it is not available to your co-authors. If this presents a problem, please contact journal staff (jp@physoc.org). Image files from the previous version are retained on the system. Please ensure you replace or remove any files that are being revised.

If you do not wish to submit a revised version of your manuscript, you must inform our journal staff (jp@physoc.org) or reply to this email to request withdrawal. Please note that a manuscript must be formally withdrawn from the peer review process at one journal before it may be submitted to another journal.

TRANSPARENT PEER REVIEW POLICY: To improve the transparency of its peer review process The Journal of Physiology publishes online, as supporting information, the peer review history of all articles accepted for publication. Readers will have access to decision letters, including Editors' comments and referee reports, for each version of the manuscript, as well as any author responses to peer review comments. Referees can decide whether or not they wish to be named on the peer review history document.

ABSTRACT FIGURES: Authors are expected to use The Journal's premium BioRender account to create/redraw their Abstract Figures. Information on how to access this account is here: https://physoc.onlinelibrary.wiley.com/journal/14697793/biorender-access.

This will enable Authors to create and download high-resolution figures. If authors have used the free BioRender service, they can use the instructions provided in the link above to download a high-resolution version suitable for publication.

The link provided should only be used for the purposes of this submission. Authors will be charged for figures created on this account if they are not related to this manuscript submission.

LANGUAGE EDITING AND SUPPORT FOR PUBLICATION: If you would like help with English language editing, or other article preparation support, Wiley Editing Services offers expert help, including English Language Editing, as well as translation, manuscript formatting, and figure formatting at www.wileyauthors.com/eeo/preparation. You can also find resources for Preparing Your Article for general guidance about writing and preparing your manuscript at www.wileyauthors.com/eeo/prepresources.

RIGOUR AND REPRODUCIBILITY: We recommend authors consult The Journal's Rigour and Reproducibility requirements to ensure that their manuscript is suitable for publication: https://physoc.onlinelibrary.wiley.com/pb-assets/hub-assets/physoc/documents/TJP-Rigour-and-Reproducibility-Requirements.pdf

REVISION CHECKLIST:

Check that your Methods section conforms to journal policy: https://jp.msubmit.net/cgi-bin/main.plex?form_type=display_requirements#methods

Check that data presented conforms to the statistics policy: https://jp.msubmit.net/cgi-bin/main.plex?form_type=display_requirements#statistics

Upload a full Response to Referees file. To create your 'Response to Referees' copy all the reports, including any comments from the Senior and Reviewing Editors, into a Microsoft Word, or similar, file and respond to each point, using font or background colour to distinguish comments and responses and upload as the required file type.

Please upload two versions of your manuscript text: one with all relevant changes highlighted and one clean version with no changes tracked. The manuscript file should include all tables and figure legends, but each figure/graph should be uploaded as separate, high-resolution files. The journal is now integrated with Wiley's Image Checking service. For further details, see: https://www.wiley.com/en-us/network/publishing/research-publishing/trending-stories/upholding-image-integrity-wileys-

image-screening-service

You may also upload:

- 'Potential Cover Art' for consideration as the issue's cover image
- Appropriate Supporting Information (Video, audio or data set: see https://jp.msubmit.net/cgi-bin/main.plex?form_type=display_requirements#supp).

We look forward to receiving your revised submission.

If you have any queries, please reply to this email and we will be pleased to advise.

Yours sincerely,

Vaughan Macefield
Senior Editor
The Journal of Physiology

REQUIRED ITEMS

1) - You must start the Methods section with a paragraph headed Ethical Approval. If experiments were conducted on humans, confirmation that informed consent was obtained, preferably in writing, that the studies conformed to the standards set by the latest revision of the Declaration of Helsinki and that the procedures were approved by a properly constituted ethics committee, which should be named, must be included in the article file. If the research study was registered (clause 35 of the Declaration of Helsinki), the registration database should be indicated, otherwise the lack of registration should be noted as an exception (e.g. The study conformed to the standards set by the Declaration of Helsinki, except for registration in a database). For further information see: https://physoc.onlinelibrary.wiley.com/hub/human-experiments.

2) - Your manuscript must include a complete Additional Information section, including competing interests; funding; author contributions and acknowledgements.

3) - Papers must comply with the Statistics Policy: https://jp.msubmit.net/cgi-bin/main.plex?form_type=display_requirements#statistics.

In summary:

- If n {less than or equal to} 30, all data points must be plotted in the figure in a way that reveals their range and distribution. A bar graph with data points overlaid, a box and whisker plot or a violin plot (preferably with data points included) are acceptable formats.

- If n > 30, then the entire raw dataset must be made available either as supporting information, or hosted on a not-for-profit repository, e.g. FigShare, with access details provided in the manuscript.

- 'n' clearly defined (e.g. x cells from y slices in z animals) in the Methods. Authors should be mindful of pseudoreplication.

- All relevant 'n' values must be clearly stated in the main text, figures and tables.

- The most appropriate summary statistic (e.g. mean or median and standard deviation) must be used. Standard Error of the Mean (SEM) alone is not permitted.

- Exact p values must be stated. Authors must not use 'greater than' or 'less than'. Exact p values must be stated to three significant figures even when 'no statistical significance' is claimed.

4) - Please include an Abstract Figure Legend within the main article file.

5) - Please ensure that all figures and tables have a title and legend, and that they have been cited within the main article text. Table 1 needs a clear title then legend.

EDITOR COMMENTS

Senior Editor:

Thank you for submitting your revised manuscript to The Journal of Physiology. It has been assessed by three independent reviewers and the Reviewing Editor has summarised what is required to allow your manuscript to be published in the Journal. We look forward to receiving your revised manuscript shortly.

In Fig 4A do not use asterisks to represent statistical significant: "(***p < 0.001, **p < 0.01)"

As per Journal policy, please report the actual p values unless p<0.001.

Reviewing Editor:

Dear authors, I'm sorry it took me a while to come up with a decision on your manuscript, especially in light of the contrary reviews. The reviewers agree that your manuscript has been improved by your revisions, however, there are some remaining changes required.

Please make sure human ethics information is complete.

Please add a figure legend for the abstract figure.

Please adjust your discussion according to the remark by reviewer 2: "As a result, I maintain that the idea that the active OFF response could represent a "clinical biomarker" of speech-relevant cognitive function is wildly over-optimistic. I also don't understand why it would be desirable to use a biomarker for SiN difficulty, when one could simply measure SiN difficulty directly. What would be the advantage to use an indirect measure, which - like SiN measurement - would require patients to not only be awake, but also be attentive?"

Please add a paragraph on how the behaving animal literature could contribute to explain the reduction of N1 in active listening as suggested by reviewer 3.

REFEREE COMMENTS

Referee #1

The manuscript in its revised version is considerably improved, especially by the addition of paired statistical testing and more comprehensive review of existing literature. I have no further concerns.

Referee #2:

Review of "Sound offset encoding is related to speech-in-noise perception at sentence level in older adults" R1

I find the manuscript somewhat improved compared to the previous version, but my main underlying concerns - that the study is underpowered and thus significant effect sizes are likely inflated, and that the study is poorly motivated - still remain.

I would therefore strongly advise the authors to dramatically tone down any claims on clinical relevance, and to be much more expansive, and more candid, about associations that they, undoubtedly, must have expected but didn't observe - particularly, the association between active OFF response size (or attentional modulation thereof) and duration discrimination threshold (DDT). If the absence of an association between OFF response size and DDT can be explained away because "the duration discrimination task likely involves additional cognitive processes, such as working memory", why should the same not apply to SiN performance? And by this logic, why should OFF responses not correlate more strongly with the word-level SiN task than the sentence-level SiN task, as the latter will involve more cognitive processes than the former? This is exactly "Why most published research findings are false" (Ioannides, PLoS Medicine, 2005): somehow, we keep finding significant effects plausible, irrespective how implausibly large they may be (see below), whilst at same time finding non-significant effects implausible, even though the respective explanations may directly contradict one another.

Anybody knowledgeable on speech-in-noise (SiN) perception will know that the idea that a unitary non-speech electrophysiological index, such as the OFF-response size, could somehow explain over 30% of SiN performance [I derived this value from the zero-order correlation coefficient, r, for the association between "Active Offset" and "SIN", which, in both the new and old versions of Table 2, is listed as r = -0.58, giving a coefficient of determination, r2, of (-0.58)^2 = 0.336, i.e., 33.6%], is outright ludicrous. I maintain that the true strength of this association - if it really exists - will be way smaller, probably more in line with the associations found between OFF response size and DDT (i.e., of the order of r ≈ -0.1, and thus r2 ≈ 0.01, or 1%, as already indicated in my first review). As a result, I maintain that the idea that the active OFF response could represent a "clinical biomarker" of speech-relevant cognitive function is wildly over-optimistic. I also don't understand why it would be desirable to use a biomarker for SiN difficulty, when one could simply measure SiN difficulty directly. What would be the advantage to use an indirect measure, which - like SiN measurement - would require patients to not only be awake, but also be attentive?

Referee #3:

The authors largely addressed all my concerns.

One remaining issue is the interpretation of the smaller N1 in active listening. The authors interpret this as a result of adaptation due to the order of task presentation, passive first then active. However, animal work on adaptation (e.g. Malmierca's group, Nelken group) suggests that significant adaptation occurs over the first few sound presentations only, and stabilizes pretty fast (e.g. Antunes et al. from the Malmierca group). while this may result in small advantage for a first block, these effects are likely to be tiny. On the other hand, recording in auditory cortex in behaving animals consistently find smaller responses in active vs. passive listening (e.g. Otazu et al. from the Zador lab, Town et al. from the Bizley lab, Bagur et al., and others). Thus, at best, the notion that adaptation is the reason for larger passive responses is weak. I would encourage the authors to review the behaving animal literature, which comes up consistently with reduction in the responses in the active condition. While the responses studied in the animal literature are not necessarily direct correlates of the N1, they are still the best we have for direct measurements of the neuronal responses during listening.

END OF COMMENTS

EDITOR COMMENTS

Senior Editor:

Thank you for submitting your revised manuscript to The Journal of Physiology. It has been assessed by three independent reviewers and the Reviewing Editor has summarised what is required to allow your manuscript to be published in the Journal. We look forward to receiving your revised manuscript shortly.

In Fig 4A do not use asterisks to represent statistical significant: "(***p < 0.001, **p < 0.01)"

As per Journal policy, please report the actual p values unless $p<0.001$.

RESPONSE: Dear editor, we would like to thank you for taking the time to review our paper and for considering it for publication in The Journal of Physiology. We are pleased to submit the revised version of our manuscript for your consideration. As advised, we have removed the asterisks in Figure 4A and reported the actual p-values, except where $p < 0.001$.

Reviewing Editor:

Dear authors, I'm sorry it took me a while to come up with a decision on your manuscript, especially in light of the contrary reviews. The reviewers agree that your manuscript has been improved by your revisions, however, there are some remaining changes required.

RESPONSE: Dear editor, we would like to thank you for taking the time to review our paper and for considering it for publication in The Journal of Physiology. We are pleased to submit a revised version of our manuscript that addresses the comments and suggestions from you and the reviewers. We believe that we have addressed the point on adaptation raised by reviewer 3, as well as all points from reviewer 2 that are directly addressable.

We would, however, like to express our concern regarding some of reviewer 2's comments, which appear to be based on assumptions that are not consistent with a scientific approach, as paraphrased below:

"Many published research findings turn out to be false, therefore research findings that I perceive to be out of line with my prior assumptions are likely to be false and therefore should not be published."

Adopting this approach would have science largely grind to a halt. Good science is about transparency, and failure to publish results is as much a problem as publication of results that might not be replicated in future.

"A correlation that appears too large to be believable at face value must instead have a 'true' correlation that is right at the lower end of what I consider plausible, and therefore inherently so small as to be irrelevant."

The reviewer is making an assumption that any correlation that could correctly be demonstrated in this context must be so small as to be irrelevant. By extension, they are saying that no correlational analyses should be attempted or reported in this field unless they support prior assumptions. Such an assumption could discourage open exploration and bias interpretation towards preconceived outcomes, which again is not consistent with good scientific practice.

As in our previous round, we have carefully addressed all reasonable and addressable comments from reviewer 2. Please find our detailed point-by-point responses below.

Please make sure human ethics information is complete.

RESPONSE: Please find below the human ethics information we have added.

'The experimental procedure was approved by the Newcastle University Research Ethics Committee, and written informed consent was obtained from all participants (reference number 46225/2023). The study conformed to the standards set by the Declaration of Helsinki (as revised in October 2013), except for registration in a database.'

Please add a figure legend for the abstract figure.

RESPONSE: We have now added a figure legend for the graphical abstract as suggested.

'Abstract Figure Legend. Schematic summary of the study investigating sound onset and offset sensitivity in the brain of older adults. EEG responses were recorded to white-noise bursts to examine neural encoding of sound onset and offset during passive listening and active task conditions. Offset responses were found to be task-dependent and influenced by attention, showing larger amplitudes during active listening. Importantly, larger active offset amplitudes were associated with better sentence-in-noise performance. These findings suggest that offset encoding reflects attention-sensitive neural processes that support speech stream parsing and real-world speech-in-noise understanding in aging listeners.'

Please adjust your discussion according to the remark by reviewer 2: "As a result, I maintain that the idea that the active OFF response could represent a "clinical biomarker" of speech-relevant cognitive function is wildly over-optimistic. I also don't understand why it would be desirable to use a biomarker for SiN difficulty, when one could simply measure SiN difficulty directly. What would be the advantage to use an indirect measure, which - like SiN measurement - would require patients to not only be awake, but also be attentive?"

RESPONSE: As you and Reviewer 2 advised, we have toned down our claim that the offset response might be a biomarker for speech-in-noise difficulty. We still believe it is important to understand the neural mechanisms underlying such listening difficulties, and a biomarker for behavioural speech-in-noise performance would be invaluable, since behavioural measures can be influenced by various confounding factors such as native language, semantic predictability, and listening effort. However, we agree that the offset response is more appropriately described as a neural substrate or neural correlate of speech-in-noise perception rather than a biomarker. Such a neural measure could indicate a specific component process of speech-in-noise difficulty, which might have distinct or complementary value to testing speech-in-noise as a whole composite process. Please find below the revised sentences in the discussion and conclusion sections.

‘Considering that sentences are more ecologically valid and more representative of real-world listening than single words (Geller et al., 2021), we propose that sound offset responses could represent a neural substrate underlying real-world listening difficulties in the population.’

‘This suggests that weak sound-offset encoding may be an underlying factor in listening difficulties, and that offset responses may reflect a neural correlate of speech-in-noise perception.’

Please add a paragraph on how the behaving animal literature could contribute to explain the reduction of N1 in active listening as suggested by reviewer 3.

RESPONSE: On the potential effect of adaptation, there is evidence showing that adaptation in the auditory cortex can occur over timescales of several minutes to tens of minutes (see the studies below).

Gill NK, Francis NA. Repetition plasticity in primary auditory cortex occurs across long timescales for spectrotemporally randomized pure tones. bioRxiv 2023.04.26.538446, 2023.

Fitzgerald K, Todd J. Hierarchical timescales of statistical learning revealed by mismatch negativity to auditory pattern deviations. Neuropsychologia 120: 25–34, 2018.

However, we agree that including findings from behaving animal studies can further support the explanation for this effect and is worth mentioning in the discussion. As you and reviewer 3 advised, we added a new paragraph to the discussion section that reviews findings from animal studies comparing active and passive listening. The added paragraph is given below.

‘The above-mentioned reduced onset responses in active listening due to adaptation mechanisms are a valid explanation that might drive this effect, since adaptation in the auditory cortex occurs over timescales ranging from milliseconds to tens of minutes (Fitzgerald & Todd, 2018; Gill & Francis, 2023; Nelken, 2014; Ulanovsky et al., 2004). Interestingly, the behaving-animal literature has reported a similar phenomenon, in which studies have shown suppressed auditory cortex activity during active listening (Otazu et al., 2009; Town, Wood, & Bizley, 2018). Dong et al. (2013) suggested that neurons in the primary auditory cortex show dual responses, with some suppressed and others enhanced by task engagement. Otazu et al. (2009) proposed that this suppression reflects an early stage of selective routing, reducing activity in neurons irrelevant to the task and thereby limiting the spread of auditory signals to other cortical and subcortical regions. In passive conditions, where no task demands are defined, auditory activity may instead propagate more broadly, enhancing the overall response to sound stimulation. The pattern observed in animal studies may resemble our human findings to some extent and could be one of the factors driving the smaller onset responses observed during active listening.’

REFEREE COMMENTS

Referee #1

The manuscript in its revised version is considerably improved, especially by the addition of paired statistical testing and more comprehensive review of existing literature. I have no further concerns.

RESPONSE: Dear reviewer, we would like to thank you for taking the time to review our paper and for helping us improve the study.

Referee #2:

Review of "Sound offset encoding is related to speech-in-noise perception at sentence level in older adults" R1

I find the manuscript somewhat improved compared to the previous version, but my main underlying concerns - that the study is underpowered and thus significant effect sizes are likely inflated, and that the study is poorly motivated - still remain.

RESPONSE: Thank you for taking the time to read our paper and share your comments. We really appreciate your helpful feedback and suggestions. Below, we address each of your points in detail.

I would therefore strongly advise the authors to dramatically tone down any claims on clinical relevance, and to be much more expansive, and more candid, about associations that they, undoubtedly, must have expected but didn't observe - particularly, the association between active OFF response size (or attentional modulation thereof) and duration discrimination threshold (DDT). If the absence of an association between OFF response size and DDT can be explained away because "the duration discrimination task likely involves additional cognitive processes, such as working memory", why should the same not apply to SiN performance? And by this logic, why should OFF responses not correlate more strongly with the word-level SiN task than the sentence-level SiN task, as the latter will involve more cognitive processes than the former? This is exactly "Why most published research findings are false" (Ioannides, PLoS Medicine, 2005): somehow, we keep finding significant effects plausible, irrespective how implausibly large they may be (see below), whilst at same time finding non-significant effects implausible, even though the respective explanations may directly contradict one another.

RESPONSE: Thank you for your comment. The expected correlation between task performance and the OFF response was a hypothesis to be tested, not a predefined outcome. The absence of such a relationship does not invalidate the hypothesis; rather, it might suggest that other factors beyond attentional engagement may contribute more strongly to behavioural performance in this task.

This interpretation is consistent with the nature of behavioural measures, which are influenced by multiple interacting processes rather than by a simple one-to-one relationship with neural indices. By proposing that additional cognitive processes might interfere with performance in the duration discrimination task, our intention was to emphasize that behaviour is shaped by many concurrent factors and cannot be reduced to a direct “2 + 2 = 4” type of logic. We do not claim that cognitive

processes alone explain the findings, but rather highlight that behavioural outcomes can vary depending on cognitive demands, task relevance, and attentional state.

Even motivation may also differ between tasks. Participants may be less engaged with the duration discrimination task (DDT) because it is relatively abstract and less intrinsically motivating, which could partly explain the larger variability observed in DDT performance. In contrast, speech-in-noise tasks are more relevant to real-world listening situations, tend to capture greater interest, and may therefore encourage more consistent attention and effort.

As you correctly noted in the previous round, offset responses depend on stimulus duration. The sound durations used in our study (1–2 s) are more comparable to the temporal structure of sentences rather than brief words. This may explain why the observed relationship appears more clearly in sentence-level SiN performance than in shorter word-level tasks.

Anybody knowledgeable on speech-in-noise (SiN) perception will know that the idea that a unitary non-speech electrophysiological index, such as the OFF-response size, could somehow explain over 30% of SiN performance [I derived this value from the zero-order correlation coefficient, r, for the association between "Active Offset" and "SIN", which, in both the new and old versions of Table 2, is listed as r = -0.58, giving a coefficient of determination, r2, of (-0.58)^2 = 0.336, i.e., 33.6%], is outright ludicrous. I maintain that the true strength of this association - if it really exists - will be way smaller, probably more in line with the associations found between OFF response size and DDT (i.e., of the order of r ≈ -0.1, and thus r2 ≈ 0.01, or 1%, as already indicated in my first review). As a result, I maintain that the idea that the active OFF response could represent a "clinical biomarker" of speech-relevant cognitive function is wildly over-optimistic. I also don't understand why it would be desirable to use a biomarker for SiN difficulty, when one could simply measure SiN difficulty directly. What would be the advantage to use an indirect measure, which - like SiN measurement - would require patients to not only be awake, but also be attentive?

RESPONSE: Thank you for clarifying the r^2 calculation. We agree that 33.6% may overestimate the actual effect size. Nevertheless, there is no evidence to suggest that this association does not exist or that it is as small as 1%, as proposed. We also believe that attributing the significant association between active offset responses and sentence-in-noise performance solely to task engagement (i.e., attention) is unlikely to be accurate. Importantly, we also observed a trend-level relationship between passive offset responses and sentence-in-noise perception ($\rho = -0.42$, $p = 0.015$). While this did not survive correction for multiple comparisons, it still suggests that offset encoding may contribute to speech-in-noise perception beyond task engagement alone. The active condition appears to strengthen this relationship rather than create it.

We therefore agree that the true effect between active offset responses and sentence-in-noise perception might be smaller than what we found. As noted in the previous revised manuscript, we have acknowledged the small sample size as a possible source of inflation in the correlation coefficient. In line with your suggestion, we have also revised the discussion and conclusion sections to use more cautious wording. Specifically, we replaced the term biomarker with neural substrate or neural correlate to more accurately describe the potential role of offset responses in speech-in-noise perception. The revised sentences are provided below.

‘Considering that sentences are more ecologically valid and more representative of real-world listening than single words (Geller et al., 2021), we propose that sound offset responses could represent a neural substrate underlying real-world listening difficulties in the population.’

‘This suggests that weak sound-offset encoding may be an underlying factor in listening difficulties, and that offset responses may reflect a neural correlate of speech-in-noise perception.’

Referee #3:

The authors largely addressed all my concerns.

One remaining issue is the interpretation of the smaller N1 in active listening. The authors interpret this as a result of adaptation due to the order of task presentation, passive first then active. However, animal work on adaptation (e.g. Malmierca's group, Nelken group) suggests that significant adaptation occurs over the first few sound presentations only, and stabilizes pretty fast (e.g. Antunes et al. from the Malmierca group). while this may result in small advantage for a first block, these effects are likely to be tiny. On the other hand, recording in auditory cortex in behaving animals consistently find smaller responses in active vs. passive listening (e.g. Otazu et al. from the Zador lab, Town et al. from the Bizley lab, Bagur et al., and others). Thus, at best, the notion that adaptation is the reason for larger passive responses is weak. I would encourage the authors to review the behaving animal literature, which comes up consistently with reduction in the responses in the active condition. While the responses studied in the animal literature are not necessarily direct correlates of the N1, they are still the best we have for direct measurements of the neuronal responses during listening.

RESPONSE: Dear Reviewer,

Thank you for taking the time to review our paper and for your comments, that have helped us to improve the manuscript. Regarding the timescales of adaptation in the auditory cortex, there is evidence showing that adaptation can occur over minutes or even tens of minutes:

Gill NK, Francis NA. Repetition plasticity in primary auditory cortex occurs across long timescales for spectrotemporally randomized pure tones. bioRxiv 2023.04.26.538446, 2023.

Fitzgerald K, Todd J. Hierarchical timescales of statistical learning revealed by mismatch negativity to auditory pattern deviations. Neuropsychologia 120: 25–34, 2018.

However, we agree that including findings from behaving animal studies can further support the explanation for this effect and is worth mentioning in the discussion. As suggested, we have added a new paragraph to the discussion section that reviews evidence from animal studies comparing active and passive listening. The added paragraph is provided below.

‘The above-mentioned reduced onset responses in active listening due to adaptation mechanisms are a valid explanation that might drive this effect, since adaptation in the auditory cortex occurs over timescales ranging from milliseconds to tens of minutes (Fitzgerald & Todd, 2018; Gill & Francis, 2023; Nelken, 2014; Ulanovsky et al., 2004). Interestingly, the behaving-animal literature has reported a similar phenomenon, in which studies have shown suppressed auditory cortex activity during active listening (Otazu et al., 2009; Town, Wood, & Bizley, 2018). Dong et al. (2013) suggested that neurons in the primary auditory cortex show dual responses, with some suppressed and others enhanced by task engagement. Otazu et al. (2009) proposed that this suppression reflects an early stage of selective routing, reducing activity in neurons irrelevant to the task and thereby limiting the spread of auditory

signals to other cortical and subcortical regions. In passive conditions, where no task demands are defined, auditory activity may instead propagate more broadly, enhancing the overall response to sound stimulation. The pattern observed in animal studies may resemble our human findings to some extent and could be one of the factors driving the smaller onset responses observed during active listening.'

Dear Dr Colak,

Re: JP-RP-2025-289104R2 "**Sound Offset Encoding is Related to Speech-in-Noise Perception at Sentence Level in Older Adults**" by Hasan Colak, Xiaoxuan Guo, Adèle Simon, Ester Benzaquén, William Sedley, Jennifer F. Linden, and Timothy D. Griffiths

Thank you for submitting your manuscript to The Journal of Physiology. It has been assessed by a Reviewing Editor and by 0 expert referees and we are pleased to tell you that it is acceptable for publication following satisfactory revision.

The referee reports are copied at the end of this email.

Please address all the points raised and incorporate all requested revisions or explain in your Response to Referees why a change has not been made. We hope you will find the comments helpful and that you will be able to return your revised manuscript within 4 weeks. If you require longer than this, please contact journal staff: jp@physoc.org.

Your revised manuscript should be submitted online using the link in your Author Tasks Link Not Available. This link is accessible via your account as Corresponding Author; it is not available to your co-authors. If this presents a problem, please contact journal staff (jp@physoc.org). Image files from the previous version are retained on the system. Please ensure you replace or remove any files that are being revised.

If you do not wish to submit a revised version of your manuscript, you must inform our journal staff (jp@physoc.org) or reply to this email to request withdrawal. Please note that a manuscript must be formally withdrawn from the peer review process at one journal before it may be submitted to another journal.

TRANSPARENT PEER REVIEW POLICY: To improve the transparency of its peer review process The Journal of Physiology publishes online, as supporting information, the peer review history of all articles accepted for publication. Readers will have access to decision letters, including Editors' comments and referee reports, for each version of the manuscript, as well as any author responses to peer review comments. Referees can decide whether or not they wish to be named on the peer review history document.

ABSTRACT FIGURES: Authors are expected to use The Journal's premium BioRender account to create/redraw their Abstract Figures. Information on how to access this account is here: https://physoc.onlinelibrary.wiley.com/journal/14697793/biorender-access.

This will enable Authors to create and download high-resolution figures. If authors have used the free BioRender service, they can use the instructions provided in the link above to download a high-resolution version suitable for publication.

The link provided should only be used for the purposes of this submission. Authors will be charged for figures created on this account if they are not related to this manuscript submission.

LANGUAGE EDITING AND SUPPORT FOR PUBLICATION: If you would like help with English language editing, or other article preparation support, Wiley Editing Services offers expert help, including English Language Editing, as well as translation, manuscript formatting, and figure formatting at www.wileyauthors.com/eeo/preparation. You can also find resources for Preparing Your Article for general guidance about writing and preparing your manuscript at www.wileyauthors.com/eeo/prepresources.

RIGOUR AND REPRODUCIBILITY: We recommend authors consult The Journal's Rigour and Reproducibility requirements to ensure that their manuscript is suitable for publication: https://physoc.onlinelibrary.wiley.com/pb-assets/hub-assets/physoc/documents/TJP-Rigour-and-Reproducibility-Requirements.pdf

REVISION CHECKLIST:

Check that your Methods section conforms to journal policy: https://jp.msubmit.net/cgi-bin/main.plex?form_type=display_requirements#methods

Check that data presented conforms to the statistics policy: https://jp.msubmit.net/cgi-bin/main.plex?form_type=display_requirements#statistics

Upload a full Response to Referees file. To create your 'Response to Referees' copy all the reports, including any comments from the Senior and Reviewing Editors, into a Microsoft Word, or similar, file and respond to each point, using font or background colour to distinguish comments and responses and upload as the required file type.

Please upload two versions of your manuscript text: one with all relevant changes highlighted and one clean version with no changes tracked. The manuscript file should include all tables and figure legends, but each figure/graph should be uploaded as separate, high-resolution files. The journal is now integrated with Wiley's Image Checking service. For further details, see: https://www.wiley.com/en-us/network/publishing/research-publishing/trending-stories/upholding-image-integrity-wileys-

image-screening-service

You may also upload:

- 'Potential Cover Art' for consideration as the issue's cover image
- Appropriate Supporting Information (Video, audio or data set: see https://jp.msubmit.net/cgi-bin/main.plex?form_type=display_requirements#supp).

We look forward to receiving your revised submission.

If you have any queries, please reply to this email and we will be pleased to advise.

Yours sincerely,

Vaughan Macefield
Senior Editor
The Journal of Physiology

EDITOR COMMENTS

Senior Editor:

Thank you for attending to the remaining concerns of the reviewers. While the Reviewing Editor is recommending acceptance, before I can make this decision you will need to submit a clean (non-highlighted) version of your manuscript without embedded figures, and with the figure legends being placed at the end of the manuscript.

Reviewing Editor:

Thank you for submitting the careful revisions of your manuscript. There are no further concerns.

END OF COMMENTS

EDITOR COMMENTS

Senior Editor:

Thank you for attending to the remaining concerns of the reviewers. While the Reviewing Editor is recommending acceptance, before I can make this decision you will need to submit a clean (non-highlighted) version of your manuscript without embedded figures, and with the figure legends being placed at the end of the manuscript.

RESPONSE: Thank you very much for taking the time to review our manuscript and for considering it for the Journal of Physiology. As you suggested, we have uploaded a clean version of the manuscript without any figures.

Reviewing Editor:

Thank you for submitting the careful revisions of your manuscript. There are no further concerns.

Dear Mr Colak,

Re: JP-RP-2025-289104R3 "**Sound Offset Encoding is Related to Speech-in-Noise Perception at Sentence Level in Older Adults**" by Hasan Colak, Xiaoxuan Guo, Adèle Simon, Ester Benzaquén, William Sedley, Jennifer F. Linden, and Timothy D. Griffiths

We are pleased to tell you that your paper has been accepted for publication in The Journal of Physiology.

The last Word (or similar) version of the manuscript provided will be used by the Production Editor to prepare your proof. When this is ready you will receive an email containing a link to Wiley's Online Proofing System. The proof should be thoroughly checked and corrected as promptly as possible.

Authors should note that it is too late at this point to offer corrections prior to proofreading. Major corrections at proof stage, such as changes to figures, will be referred to the Editors for approval before they can be incorporated. Only minor changes, such as to style and consistency, should be made at proof stage. Changes that need to be made after proof stage will usually require a formal correction notice.

All queries at proof stage should be sent to: TJP@wiley.com.

Following copyediting and typesetting the accepted version will be published online.

Yours sincerely,

Vaughan Macefield
Senior Editor
The Journal of Physiology

IMPORTANT POINTS TO NOTE FOLLOWING ACCEPTANCE OF YOUR PAPER:

• IMPORTANT NOTICE ABOUT OPEN ACCESS: To assist authors whose funding agencies mandate immediate public access to published research findings, The Journal of Physiology allows authors to pay an Open Access (OA) fee to have their papers made freely available immediately on publication.

The Corresponding Author will receive an email from Wiley with details on how to register or log-in to Wiley Authors Services where you will be able to place an order.

• You can check if your funder or institution has a Wiley Open Access Account here: https://authorservices.wiley.com/author-resources/Journal-Authors/licensing-and-open-access/open-access/author-compliance-tool.html.

• TRANSPARENT PEER REVIEW POLICY: To improve the transparency of its peer review process, The Journal of Physiology publishes online as supporting information the peer review history of all articles accepted for publication. Readers will have access to decision letters, including Editors' comments and referee reports, for each version of the manuscript as well as any author responses to peer review comments. Referees can decide whether or not they wish to be named on the peer review history document.

• You can help your research get the attention it deserves! Check out Wiley's free Promotion Guide for best-practice recommendations for promoting your work at: www.wileyauthors.com/eeo/guide. You can learn more about Wiley Editing Services which offers professional video, design, and writing services to create shareable video abstracts, infographics, conference posters, lay summaries, and research news stories for your research at: www.wileyauthors.com/eeo/promotion.

• If you would like to receive our 'Research Roundup', a monthly newsletter highlighting the cutting-edge research published in The Physiological Society's family of journals (The Journal of Physiology, Experimental Physiology, Physiological Reports, The Journal of Nutritional Physiology and The Journal of Precision Medicine: Health and Disease), please click this link, fill in your name and email address and select 'Research Roundup':
https://www.physoc.org/journals-and-media/membernews

EDITOR COMMENTS

Senior Editor:

Thank you, I am pleased to report that your manuscript is now considered acceptable for publication in The Journal of Physiology.
